# Effect of Omega-3 Fatty Acid Intake on Circulating Biomarkers of Atrial Fibrillation-Related Pathways in the PREDIMED-Plus Study

**DOI:** 10.3390/nu18111669

**Published:** 2026-05-23

**Authors:** Jaime Lara Moreno, Linzi Li, Alvaro Alonso, Dora Romaguera, Angel M. Alonso-Gómez, Cristina Razquin, Lucas Tojal-Sierra, Miquel Fiol, Miguel A. Martinez-Gonzalez, Vinita Subramanya, Jordi Salas-Salvadó, Montserrat Fitó, Estefanía Toledo

**Affiliations:** 1 Department of Preventive Medicine and Public Health, University of Navarra, Calle Irunlarrea 1, 31008 Pamplona, Spain; jlaramoreno@alumni.unav.es (J.L.M.); crazquin@unav.es (C.R.); mamartinez@unav.es (M.A.M.-G.); 2 Department of Epidemiology, Rollins School of Public Health, Emory University, 1518 Clifton Road NE, Atlanta, GA 30322, USA; linzi.li@emory.edu (L.L.); alvaro.alonso@emory.edu (A.A.); vinita.subramanya@emory.edu (V.S.); 3 Centro de Investigación Biomédica en Red de Fisiopatología de la Obesidad y Nutrición (CIBEROBN), Instituto de Salud Carlos III (ISCIII), Avenida Monforte de Lemos, 5, 28029 Madrid, Spain; mariaadoracion.romaguera@idisba.es (D.R.); angelmago13@gmail.com (A.M.A.-G.); lutojal@hotmail.com (L.T.-S.); jordi.salas@urv.cat (J.S.-S.); mfito@researchmar.net (M.F.); 4 Health Research Institute of the Balearic Islands (IdISBa), Carretera de Valldemossa, 79, Edificio S, 07120 Palma de Mallorca, Spain; mfiolsala@gmail.com; 5 Bioaraba Health Research Institute, Araba University Hospital, University of the Basque Country UPV/EHU, Carrer de Jose Atxotegi, s/n, 01009 Vitoria-Gasteiz, Spain; 6 Navarra Institute for Health Research (IdiSNA), Calle Irunlarrea 3, 31008 Pamplona, Spain; 7 Department of Nutrition, Harvard T. H. Chan School of Public Health, Harvard University, 677 Huntington Avenue, Boston, MA 02115, USA; 8 Human Nutrition Unit, Department of Biochemistry and Biotechnology, Institut d’Investigacions Sanitàries Pere i Virgili, Rovira i Virgili University, Carrer de Sant Llorenç, 21, 43201 Reus, Spain; 9 Hospital del Mar Medical Research Institute (IMIM), Carrer de Doctor Aiguader, 88, 08003 Barcelona, Spain

**Keywords:** omega-3 fatty acids, atrial fibrillation, Mediterranean diet, PREDIMED-Plus

## Abstract

**Background/Objectives:** Whether habitual dietary omega-3 fatty acid intake is reflected in circulating biomarkers of atrial fibrillation (AF)-related pathways is unclear. We assessed whether usual dietary intake of *n*-3 fatty acids—considered as total, marine-derived, or non-marine-derived—was associated with the trajectories of five serum markers that reflect AF-related mechanistic pathways [*N*-terminal pro-B-type natriuretic peptide (NT-pro-BNP), high-sensitivity troponin T (hs-TnT), high-sensitivity *C*-reactive protein (CRP), the *C*-terminal propeptide of type-I procollagen (PICP), and 3-nitrotyrosine (3-NT)] over 5 years of follow-up. **Methods:** In 510 participants of the PREDIMED-Plus trial (older Spanish adults with metabolic syndrome), we measured plasma NT-pro-BNP, hs-TnT, CRP, PICP, and 3-NT at baseline and after 3 and 5 years. Energy-adjusted omega-3 intake was assessed with a validated 143-item food-frequency questionnaire. Cross-sectional and 5-year longitudinal associations according to tertiles of omega-3 fatty acid intake were estimated with linear regression and mixed-effects models. **Results:** Median total omega-3 intake was 2.0 g/day. Total omega-3 intake was not associated with any biomarker, neither cross-sectionally nor longitudinally. Marine omega-3 was directly associated cross-sectionally with 3-NT (highest vs. lowest tertile +28.4%, 95% CI 5.5 to 56.2; *p*-trend = 0.014) but not longitudinally. Moderate baseline non-marine omega-3 fatty acid intake was associated with a decrease in PICP after 5 years of follow-up. **Conclusions:** Overall, habitual total omega-3 fatty acid intake was not associated with circulating AF-related pathways. The sporadic association between marine omega-3 fatty acid intake and 3-NT in the cross-sectional assessment and the isolated non-linear association between baseline non-marine omega-3 fatty acid intake and PICP after 5 years warrant further investigation.

## 1. Introduction

Atrial fibrillation (AF) is the most prevalent sustained cardiac arrhythmia in adults and a leading cause of stroke, heart failure, and all-cause mortality [1,2]. The global age-standardized prevalence reached approximately 60 million cases in 2019, representing a near-doubling over the preceding three decades, and the number of affected adults is projected to double again over the next 30 years. AF is associated with an approximately five-fold increase in stroke risk, an approximately 1.5- to 1.9-fold increase in all-cause mortality (greater in women than in men), and a substantial burden of hospitalization and heart failure, underscoring the clinical relevance of primary-prevention strategies addressing modifiable dietary exposures [3].

The onset and perpetuation of AF follow a process of atrial remodeling that is typically initiated by focal ectopic triggers arising within the atrial tissue [4]. Progressive electrical and structural changes gradually sustain the arrhythmia and lead to macroscopic enlargement of the atria [5,6]. Indeed, dilation of the left atrium is recognized both as a fundamental feature of the remodeling process and as an independent prognostic indicator of incident AF [7]. A cluster of comorbid conditions—arterial hypertension, type-2 diabetes, obesity, sleep-related breathing disorders, tobacco exposure, and renal impairment—accelerate these fibrotic and dilatory changes and thereby elevate AF risk [7]. Chronic inflammatory activation additionally plays a pivotal role in both triggering and maintaining the arrhythmia.

Five complementary plasma biomarkers reflect distinct mechanistic axes underlying AF: NT-pro-BNP indexes ventricular wall stretch and atrial loading; hs-TnT reflects subclinical cardiomyocyte injury; CRP captures low-grade systemic inflammation; PICP marks type-I procollagen synthesis and atrial fibrosis; and 3-NT reflects peroxynitrite-mediated oxidative protein modification [8,9]. Together, these analytes serve as indirect mechanistic surrogates of the principal pathways implicated in AF emergence and progression.

Long-chain *n*-3 polyunsaturated fatty acids—particularly eicosapentaenoic acid (EPA) and its plant precursor alpha-linolenic acid (ALA)—have attracted interest as potential modifiers of AF risk. The endogenous conversion of plant-sourced ALA, found in plant-based foods, must be converted into EPA, which predominates in marine foods, is limited in humans and proceeds with low efficiency [10]. Once integrated into cardiomyocyte phospholipids, EPA modulates membrane composition and exerts anti-inflammatory actions, including suppression of cytokine signaling [11]. An additional target of interest is the mechanosensitive Piezo1 channel, through which *n*-3 species may influence atrial electrogenesis [12]. Crucially, the net effect appears non-monotonic with dose: modest intakes may be cardioprotective, whereas supraphysiological amounts have been linked to pro-arrhythmic responses.

The epidemiological and clinical literature has produced inconsistent signals regarding *n*-3 fatty acids and AF. Among Danish men, displacing saturated fat with *n*-3 was linked to greater arrhythmic incidence [13]. Pooled analyses of randomized trials have likewise described elevated AF occurrence when marine-derived supplements exceeded 1 g/day [14], a signal reinforced by the RESPECT-EPA trial with long-term eicosapentaenoic acid supplementation at 1.8 g/day [15]. By contrast, brief peri-cardioversion courses of omega-3 have been tied to fewer recurrences [16], and similar short-term perioperative use has shown protective trends against postoperative AF [17,18]. Plant-derived ALA—abundant in walnuts, flaxseed, and some vegetable oils—partially elongates to EPA and may exert distinct effects on the same pathways. Divergent biological effects across food-based and pharmacological sources complicate the interpretation of these findings, and few studies have jointly examined dietary marine and non-marine omega-3 sources in relation to AF-related pathways in older adults at high cardiovascular risk.

Thus, in a biomarker substudy nested within the Spanish PREDIMED-Plus trial, we examined cross-sectional and 5-year longitudinal associations between habitual dietary intake of *n*-3 fatty acids—considered as total, marine-derived, or non-marine-derived—and five serum biomarkers reflecting AF-related mechanistic pathways (NT-pro-BNP, hs-TnT, CRP, PICP, and 3-NT).

## 2. Materials and Methods

### 2.1. Study Participants

The PREDIMED-Plus study is an ongoing multicenter randomized trial enrolling 6874 adults (men aged 55–75 years and women aged 60–75 years) with overweight or obesity (BMI 27.0–<40.0 kg/m^2^) and metabolic syndrome [19,20]. Recruitment proceeded from 2013 through 2016 across 23 Spanish centers and comprised 3574 men and 3300 women. Eligible participants had a body mass index (BMI) between 27 and <40 kg/m^2^ and met at least three components of the metabolic syndrome. Individuals with prevalent AF, any prior cardiovascular event at screening, or recent (previous 5 years) malignancy were ineligible. Participants were randomized in a 1:1 ratio to an intensive lifestyle intervention (ILI) combining an energy-restricted Mediterranean diet (erMedDiet), physical-activity promotion, and cognitive–behavioral support, or to a control arm receiving general advice on the traditional Mediterranean diet without caloric restriction. Both arms received free extra-virgin olive oil (1 L/month). The active intervention lasted for 6 years, with 2 additional years of follow-up, for outcome ascertainment. The trial is registered at www.isrctn.com/ISRCTN89898870 (registered on 24 July 2014; accessed on 7 April 2026), and the full protocol is available at www.predimedplus.com/en/project/ (accessed on 7 April 2026) [19,21]. Although PREDIMED-Plus is a randomized trial, the present analysis is observational because the exposure of interest (dietary omega-3 intake) was not randomized.

**Figure 1:**
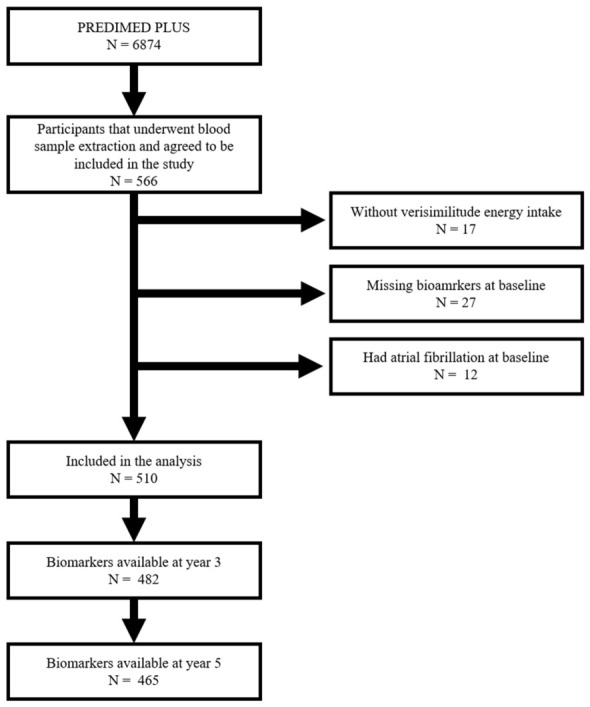
Flow chart of study participants.

This biomarker substudy comprised 510 participants recruited at three sites—University Clinic of Navarra, Araba University Hospital, and Son Espases University Hospital—for whom serum samples were available at baseline and at the 3-year and 5-year visits. The biomarker substudy was restricted to three centers on the basis of local laboratory capability and logistical feasibility for acquisition of echocardiograms; participants were selected consecutively among those attending the baseline clinic visit who agreed to participate in the substudy. From the source population of the three sites (*n* = 566), we excluded participants with implausible daily energy intakes (<500 or >3500 kcal/day in men, <800 or >4000 kcal/day in women; *n* = 17), with all five baseline biomarkers missing (*n* = 27), and with prevalent AF at baseline (*n* = 12), yielding the analytical sample of 510 (Figure 1). Current analyses were conducted based on a dataset with follow-up information up to 5 years, generated on 10 August 2022.

### 2.2. Dietary Assessment

Habitual diet was assessed at each visit by trained dieticians using a previously validated 143-item semi-quantitative food-frequency questionnaire (FFQ) [22]. The FFQ assessed the dietary consumption over the previous year and included 9 prespecified frequency categories of consumption ranging from “never or seldom” to “≥6 times/day”. Daily food intake was estimated by multiplying the reported frequency by the prespecified serving size. Total energy intake and macro- and micronutrients were estimated from Spanish food-composition tables [23,24]. Adherence to the Mediterranean diet was assessed with a 17-item screener of adherence to an erMedDiet developed within the frame of the PREDIMED-Plus trial [25].

Dietary omega-3 fatty acid intake was operationalized in three pre-specified ways: (i) total omega-3—α-linolenic acid (ALA, C18:3 *n*-3) + eicosapentaenoic acid (EPA, C20:5 *n*-3) + docosapentaenoic acid (DPA, C22:5 *n*-3) + docosahexaenoic acid (DHA, C22:6 *n*-3), in g/day, our principal exposure; (ii) marine omega-3—EPA + DPA + DHA from fish and shellfish, with the minor contribution of marine ALA assumed negligible; and (iii) non-marine omega-3—predominantly ALA from plant foods (walnuts and other tree nuts, and vegetable oils such as walnut oil) plus a minor contribution from non-marine animal sources (poultry, eggs, dairy), computed as total minus marine. Marine-derived omega-3 fatty acid intake (EPA, DPA, DHA) was estimated from eight FFQ items capturing the primary dietary sources of long-chain omega-3 fatty acids in the Spanish diet: (1) white fish—hake, sole, sea bream, monkfish; (2) oily fish—sardines, tuna, bonito, mackerel, salmon; (3) salted fish—cod, salt-cured fish; (4) bivalve mollusks—oysters, clams, mussels; (5) cephalopods—squid, octopus, baby squid, cuttlefish; (6) crustaceans—prawns, king prawns, lobster; (7) canned seafood in brine—sardines, anchovies, bonito, tuna; and (8) canned seafood in oil—sardines, anchovies, bonito, tuna. All three exposures were energy-adjusted by the residual method, separately by sex, and categorized into tertiles [26].

### 2.3. Measurement of Biomarkers

At each visit, peripheral venous blood samples after an overnight fast were obtained at baseline (*n* = 510), 3 years (*n* = 482), and 5 years (*n* = 465) of follow-up. Samples were processed within four hours and stored at –80 °C. We measured the concentration of the following biomarkers: NT-pro-BNP—as a biomarker for atrial stretch—and hs-TnT—as biomarker for myocardial damage—using electrochemiluminescence assay (ECLIA) on the Cobas 8000 Analyzer module e602 (Roche Diagnostics, Basel, Switzerland); high-sensitivity CRP—as a biomarker for systemic inflammation—using latex-enhanced immunoturbidimetry on a Cobas 8000 autoanalyzer (Roche Diagnostics, Mannheim, Germany); PICP—as a biomarker for cardiac fibrosis—using competitive enzyme immunoassay (MicroVue PICP EIA, Quidel, San Diego, CA, USA); and 3-NT—as biomarker of nitrosative/oxidative stress—using competitive ELISA (human Nitrotyrosine ELISA kit, Abcam, Cambridge, UK). All samples from the same participant were analyzed together in the same batch with laboratory personnel blinded to exposure and clinical data, thus avoiding batch effects. Biomarker concentrations were natural-log transformed prior to modeling.

### 2.4. Assessment of Covariates

At enrolment, a structured questionnaire captured demographic and social information on sociodemographic characteristics (age, sex, civil status, attained education), and personal medical history (type-2 diabetes, dyslipidemia, arterial hypertension, and obstructive sleep apnea [OSA]). Certified personnel obtained anthropometric data (height and weight) and measured systolic/diastolic blood pressure under standardized conditions. BMI was computed as the ratio of weight in kilograms to the square of height in meters. Renal function was indexed by eGFR estimated via the creatinine-based CKD-EPI 2021 equation [27]; creatinine itself was determined using the Jaffe methodology.

### 2.5. Statistical Analyses

Baseline characteristics were described according to the baseline tertiles of total omega-3 fatty acid intake and were expressed as mean (standard deviation) for the continuous variables and as percentages for categorical variables. Baseline characteristics of the analytic sample (*n* = 510) were compared to those of the overall PREDIMED-Plus cohort.

All biomarkers were natural-log transformed to improve normality; results were back-transformed and reported as percent differences calculated with the equation [exp(β) − 1] × 100%.

Cross-sectional associations were estimated from multivariable linear regression models to assess the cross-sectional association between baseline tertiles of log-transformed baseline biomarker and the omega-3-intake tertile variable. The p for trend was estimated by assigning the tertile-specific median to participants in each tertile and treating the resulting variable as quantitative in the regression models. Longitudinal associations between baseline tertiles of total omega-3 fatty acid intake and 5-year changes in the log-transformed blood-based biomarkers were assessed with linear mixed-effects models with random intercepts at the participant level and an unstructured covariance structure. Time was modeled as a categorical variable at baseline, year 3, and year 5, and visit-by-exposure interactions were explicitly tested. Models were adjusted for sex, age, marital status (4 categories), educational level, prevalent diabetes, prior cancer, smoking status (3 categories), prevalent dyslipidemia, prevalent hypertension, previous history of OSA, physical activity, BMI, eGFR (CKD-EPI 2021), total energy intake, and adherence to the erMedDiet. Missing repeated outcome data were handled under a missing-at-random assumption using all available observations through maximum-likelihood estimation.

Parallel analyses were additionally conducted using time-updated intake for omega-3 exposure, relying on the biomarkers using repeated dietary assessments obtained at the 3- and 5-year visits. For models with time-updated omega-3 exposure, other dietary variables, physical activity and eGFR were also updated.

Marine and non-marine intakes were examined individually, and in these analyses, the two exposures were additionally adjusted for each other. Two-sided *p*-values < 0.05 were considered statistically significant. Statistical computations were executed in Stata v.16.0 SE (StataCorp LLC, College Station, TX, USA).

### 2.6. Sensitivity Analyses: Methods

Sensitivity analyses included sequential exclusion of BMI and/or the 17-item Mediterranean-adherence score from the adjustment set, to assess potential overadjustment by mediators of the diet-biomarker pathway. To address potential overadjustment by BMI and erMedDiet adherence, we refitted the cross-sectional and longitudinal primary models under four specifications: fully adjusted (primary), no-BMI adjusted, no-erMedDiet adjusted, and neither-BMI-nor-erMedDiet adjusted.

In addition, inverse-probability-of-attrition weighting (IPAW) using stabilized weights derived from baseline predictors of dropout, and a canonical cumulative Robins–Hernán formulation for the non-marine omega-3 × NT-pro-BNP signal, was used to formally assess potential attrition bias at years 3 (*n* = 482) and 5 (*n* = 465). Also, baseline characteristics of retained versus lost participants were compared using standardized mean differences.

Also, we complemented our main tertile-based analyses with restricted cubic splines using the continuous exposure to describe in more detail the non-linear association between baseline non-marine omega-3 fatty acid intake and changes in PICP over time. Knots were placed at the 5th, 35th, 65th and 95th percentiles of the non-marine omega-3 distribution. As a sensitivity analysis, we compared the restricted cubic splines with 4 knots to models with 3 or 5 knots using the Akaike information criterion.

### 2.7. Ethics

Research Ethics Committees of all participating centers approved the parent study protocol (registration ISRCTN89898870), and all participants provided written informed consent. The biomarker secondary analysis was approved by the Emory University Institutional Review Board (IRB00100921) and by the local ethics committees of the three participating PREDIMED-Plus sites.

## 3. Results

### 3.1. Population Characteristics

Baseline characteristics of the 510 participants by tertile of total omega-3 intake are shown in Table 1. Median total omega-3 intake was 2.00 g/day (tertile cut-points 1.76 and 2.37 g/day), of which marine sources contributed roughly one quarter on average (median 0.5 g/day). Compared to participants in the lowest tertile, those participants in the highest tertile of total omega-3 intake were less likely to be current smokers, reported higher physical activity, adhered more strongly to the Mediterranean diet, and were less likely to have prevalent diabetes, hypertension, or OSA. Mean baseline biomarker concentrations did not markedly differ across tertiles (Table 1).

### 3.2. Cross-Sectional Associations

**Figure 2:**
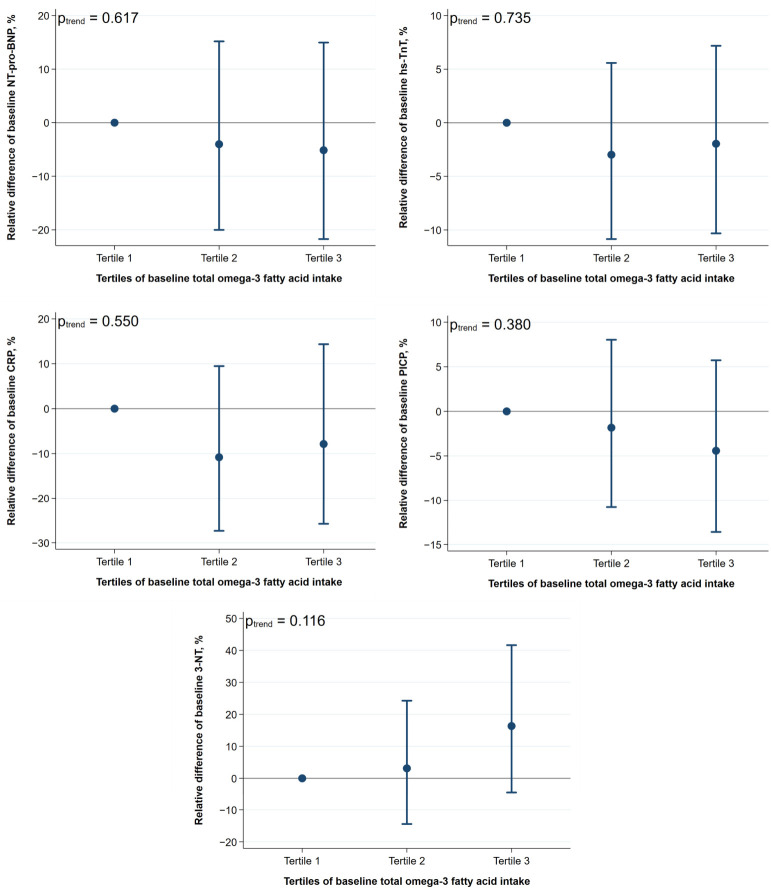
Cross-sectional association between tertiles of baseline total omega-3 fatty acid intake and baseline biomarkers of atrial fibrillation-related pathways. Multivariable model: adjusted for sex, age, intervention group, recruitment center, prevalent diabetes, previous history of cancer, smoking habit at baseline (3 categories), civil status, prevalent dyslipidemia, prevalent hypertension, previous history of sleep apnea, eGFR, time of physical activity, BMI, total caloric intake, educational level, and adherence to an energy-reduced Mediterranean diet.

After multivariable adjustment, baseline concentrations of the biomarkers did not differ across tertiles of total omega-3 intake (Figure 2). When omega-3 intake was decomposed into marine and non-marine sources, higher marine omega-3 fatty acid intake was associated with higher concentrations of 3-NT, whereas non-marine omega-3 showed no cross-sectional association with 3-NT or any other biomarker (Figure 2; Supplemental Figures S1 and S2).

### 3.3. Longitudinal Trajectories

Retention was high (94.5% at year 3 and 91.2% at year 5), and no substantial differences were observed between retained participants and those with missing biomarker information at a given follow-up visit (Supplemental Table S3).

**Figure 3:**
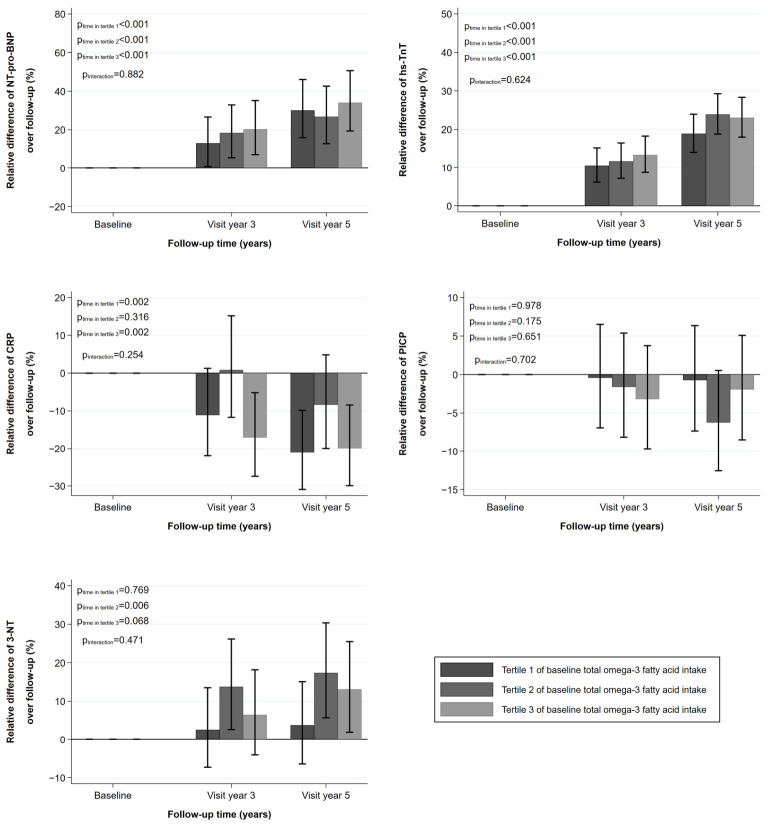
Association between baseline total omega-3 fatty acid intake and biomarkers of atrial fibrillation-related pathways over follow-up. Multivariable model: adjusted for sex, age, intervention group, recruitment center, prevalent diabetes, previous history of cancer, smoking habit at baseline (3 categories), civil status, prevalent dyslipidemia, prevalent hypertension, previous history of sleep apnea, eGFR, time of physical activity, BMI, total caloric intake, educational level, and adherence to an energy-reduced Mediterranean diet.

**Figure 4:**
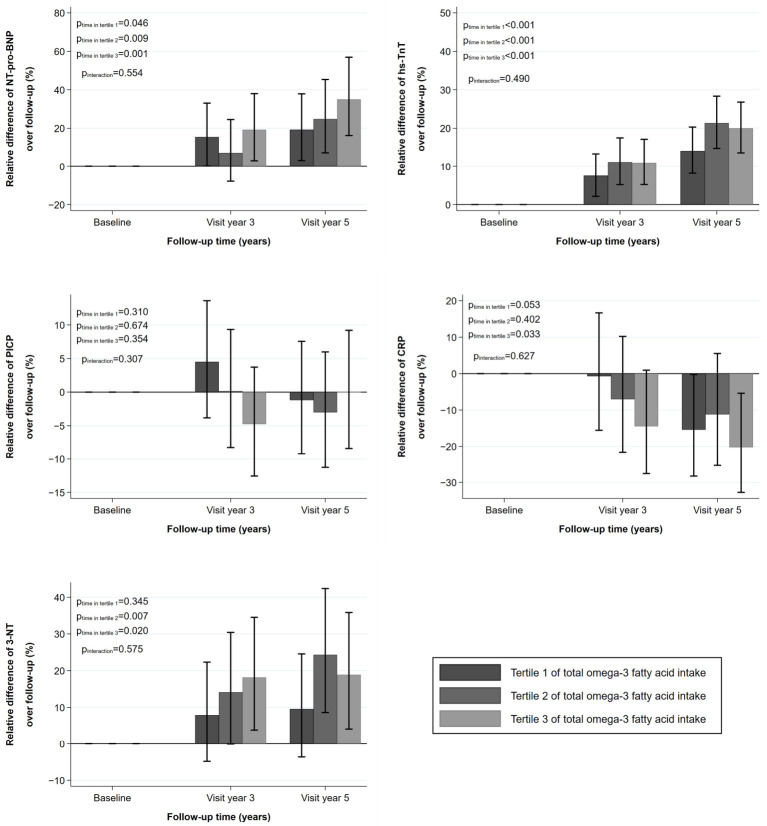
Association between updated total omega-3 fatty acid intake and biomarkers of atrial fibrillation-related pathways over follow-up. Multivariable model: adjusted for sex, age, intervention group, recruitment center, prevalent diabetes, previous history of cancer, smoking habit at baseline (3 categories), civil status, prevalent dyslipidemia, prevalent hypertension, previous history of sleep apnea, eGFR, time of physical activity, BMI, total caloric intake, educational level, and adherence to an energy-reduced Mediterranean diet.

Biomarkers, except for PICP, showed background change over 5 years of follow-up; however, no statistically significant tertile-by-visit interaction was detected for the associations between total omega-3 fatty acid intake and any biomarker, neither for the baseline-fixed exposure (Figure 3) nor for the time-updated exposure (Figure 4). Marine omega-3 intake was not associated with significant between-tertile differences over time (Supplemental Figures S3 and S5). Moderate baseline non-marine omega-3 fatty acid intake was associated with a decrease in PICP after 5 years of follow-up (Supplemental Figure S4). This latter association was not observed with updated intake of non-marine omega-3 fatty acid intake over follow-up (Supplemental Figure S6).

### 3.4. Sensitivity Analyses: Results

Sensitivity analyses that successively excluded BMI, erMedDiet adherence, or both from the adjustment set barely changed the results (Supplemental Table S2a–f). The inverse-probability-of-attrition-weighted longitudinal estimates were materially indistinguishable from the unweighted primary estimates (Supplemental Table S3a–c). Restricted cubic splines using baseline non-marine omega-3 as exposure and PICP over time as the dependent variable showed a non-monotonic association (Supplemental Figure S7). Comparison to models with 3 or 5 knots yielded qualitatively identical results.

## 4. Discussion

In this 5-year follow-up of 510 Spanish older adults at high cardiovascular risk, habitual total dietary omega-3 fatty acid intake was not associated with circulating biomarkers of atrial wall stretch (NT-pro-BNP), cardiomyocyte injury (hs-TnT), systemic inflammation (CRP), or collagen turnover (PICP), although a non-linear (J-shaped) trajectory of PICP over 5 years across the range of non-marine omega-3 intake emerged in spline analyses. Marine omega-3 intake was associated cross-sectionally with higher 3-NT concentrations, but not with longitudinal trajectories of any biomarker. Moderate updated non-marine omega-3 fatty acid intake was not associated with increases in NT-pro-BNP at visit 3, but it was at visit 5 (Supplemental Figure S6).

Evidence from omega-3 supplementation trials remains heterogeneous. Pharmacological doses above 3 g/day of EPA or DHA have been tied to higher AF occurrence, as illustrated by the REDUCE-IT [28] and STRENGTH [29] trials. With more moderate doses (around 1 g/day), individual trials—ASCEND [30], VITAL [31], and GISSI-HF [32]—did not flag a clear increase in AF, although their pooled estimate pointed to a modest 12% relative excess [14]. The RESPECT-EPA trial subsequently reported a substantive absolute-risk increment for new-onset AF among volunteers receiving 1.8 g/day of EPA compared to placebo [15]. It deserves emphasis that only VITAL and GISSI-HF captured dietary intake, whereas the remaining trials quantified circulating EPA concentrations. The habitual marine intake in our PREDIMED-Plus subsample—0.72 g/day on average, ranging from 0.41 g/day in the lowest tertile to 1.10 g/day in the highest—remained substantially below the supplemental doses tested in the trials, where AF risk has been most consistently elevated at and above 1.8 g/day. A recent pooled analysis contrasted food-derived and pharmaceutical sources of omega-3 and documented opposing directions of effect: greater dietary omega-3 fatty acid intake predicted a lower AF incidence, whereas pharmacological dosing elevated AF risk in a dose-dependent fashion [33].

Three mechanistic pathways plausibly link omega-3 fatty acids to AF biology. First, EPA and DHA incorporate into sarcolemmal phospholipids and modulate cardiac ion currents, potentially reducing arrhythmogenic susceptibility [34]. Second, they may enhance mitochondrial fatty-acid β-oxidation in atrial myocytes, which are energetically vulnerable during rapid atrial rates [35]. Third, they serve as substrates for pro-resolving lipid mediators and attenuate NF-κB-driven inflammatory signaling implicated in atrial fibrosis [36,37]. We did not measure atrial-specific inflammatory cytokines (e.g., IL-1β, IL-6); hs-CRP was included as a systemic inflammatory proxy, which is a limitation we explicitly acknowledge.

The cross-sectional positive association between marine omega-3 fatty acid intake and 3-NT—a marker of peroxynitrite-modified protein—should not be interpreted as causal. Several non-causal explanations are more plausible than a direct pro-oxidative effect of marine omega-3 intake: (i) reverse confounding by oily fish recommendations preferentially adopted by participants with prior cardiovascular events or higher oxidative load; (ii) shared variance with other dietary components plausibly affecting 3-NT (e.g., heme iron, methylmercury, advanced glycation end-products from cooking); and (iii) chance in the context of multiple testing. Indeed, Kusunoki and colleagues showed that omega-3 PUFAs induce heme oxygenase-1 expression in adipocyte cultures, a cytoprotective response that curtails the formation of reactive nitrogen species—including peroxynitrite, the upstream precursor of 3-NT—supporting an antioxidant rather than pro-oxidant role for omega-3 species [38]. The absence of a longitudinal signal across 5 years of follow-up, together with the well-established antioxidant properties of long-chain omega-3 fatty acids in interventional studies, argues strongly against a causal interpretation of this isolated baseline finding.

Previous population studies generally describe an inverse relation between circulating omega-3 fatty acids and CRP. Among middle-aged Finnish men, elevated serum omega-3 paralleled reduced circulating CRP [39]. Micallef and colleagues similarly reported that plasma EPA and DPA concentrations were inversely associated with CRP in healthy volunteers [40]. A French observational analysis by Julia et al. linked higher dietary PUFAs intake (both *n*-3 and *n*-6) to lower long-term CRP values, with vitamin E intake behaving as an effect modifier [41]. A meta-analytic synthesis by Pan et al. further suggested a CRP-lowering action of supplemental omega-3 in oncology patients [42]. These inverse associations were not replicated in our Mediterranean subsample, possibly because the baseline anti-inflammatory tone of the Mediterranean dietary pattern leaves little residual variance for omega-3 intake to explain.

A separate population-based investigation in Eastern Finnish men explored circulating omega-3 fatty acids in relation to plasma NT-pro-BNP and identified DPA as the only omega-3 subspecies with an inverse association, which persisted after adjustment for age and cardiovascular risk factors [43]. Mechanistically, BNP has been shown to antagonize TGF-β signaling within cardiac fibroblasts, contributing to antifibrotic effects at the myocardial level [44]. Through this cross-talk with natriuretic-peptide pathways, omega-3 species could plausibly support cardiovascular homeostasis in ways relevant to atrial pathology.

With regard to collagen turnover, a non-linear association between non-marine omega-3 intake and 5-year change in PICP was observed. Intakes within the central range (≈1.3–1.7 g/day) were associated with a modest reduction in this collagen-synthesis marker, with values returning toward baseline at the highest intakes (with wider confidence intervals). This non-monotonic pattern, only partially captured by the tertile analysis, suggests that habitual non-marine (largely ALA-rich) omega-3 intake within the usual dietary range may attenuate type I procollagen turnover, while very low intakes flag a coexisting profile of accelerated extracellular-matrix remodeling.

Among non-marine sources, walnuts may be particularly relevant: regular consumption raises circulating ALA and, modestly, EPA, and may favorably reshape the systemic *n*-6/*n*-3 ratio [45]. Whether these changes underlie the non-monotonic PICP signal observed at moderate non-marine intakes cannot be established from our data and warrants targeted investigation.

Translating these biomarker findings into AF risk implications requires caution. The five markers studied are mechanistic surrogates rather than validated intermediate endpoints for AF: elevated NT-pro-BNP and hs-TnT have been associated with incident AF in prospective cohorts, while PICP and 3-NT reflect upstream remodeling processes whose quantitative link to AF onset is less well characterized. The overall absence of longitudinal associations therefore suggests that, within the dietary range observed in this Mediterranean cohort, habitual omega-3 intake is unlikely to shape AF risk through these specific pathways—although effects via mechanisms not captured here (e.g., direct electrophysiological modulation, vagal tone) cannot be excluded.

The present analysis has several notable strengths. First, the breadth of individual-level data gathered throughout the parent trial enabled simultaneous adjustment for an extensive panel of covariates. Moreover, retention was excellent, limiting the likelihood that attrition-driven selection substantially distorted the estimates. In addition, the five-year follow-up offered ample time for biomarker trajectories to become detectable. Also, all serum determinations from a given volunteer were processed together, eliminating inter-run drift. Finally, repeated dietary assessments in the setting of a Mediterranean lifestyle intervention—where marine and non-marine omega-3 sources were actively promoted—enabled us to operationalize time-updated exposure as an additional analytic strategy.

A number of limitations temper the interpretation of our results. First, although PREDIMED-Plus is a randomized trial, dietary omega-3 intake was not itself randomized; the observed associations therefore remain susceptible to residual confounding by lifestyle factors that differ across tertiles—smoking, physical activity, Mediterranean diet adherence, and the prevalence of diabetes, hypertension and OSA, as shown in Table 1. Despite multivariable adjustment for these covariates, unmeasured or imperfectly measured confounders may persist, and causal inference cannot be drawn from these observational analyses. Second, exposure rests on self-reported information; although the FFQ had previously demonstrated acceptable validity [22], some measurement error is inevitable, and this misclassification is expected to attenuate associations toward the null. Third, the study population—older adults with abdominal obesity and metabolic syndrome—narrows the external generalizability of our conclusions. Fourth, the sample size, while adequate for moderate-sized effects, may be underpowered for small ones. Fifth, we focused on circulating analytes, which should be interpreted as indirect mechanistic surrogates of AF-related pathways (atrial stretch, myocardial injury, systemic inflammation, cardiac fibrosis, and nitrosative stress) rather than hard clinical endpoints (incident AF, stroke, or cardiovascular mortality); these hard outcomes will warrant future analyses once adjudicated data accumulate in the cohort. Sixth, the isolated cross-sectional association between higher omega-3 intake and higher 3-NT is biologically counterintuitive given the antioxidant properties of long-chain omega-3 fatty acids, and most plausibly reflects residual confounding, chance in the context of multiple testing, or assay noise; it should therefore not be over-interpreted, and the longitudinal analyses—which are the primary aim of this work—did not reproduce this baseline signal. Seventh, residual confounding by overall Mediterranean dietary pattern adherence is difficult to separate entirely from omega-3 intake despite our adjustments. Comparisons with supplement trials (such as VITAL, OMEMI, STRENGTH, and REDUCE-IT) or with studies of circulating omega-3 biomarkers should therefore be interpreted cautiously because dose, bioavailability, and causal interpretability differ materially from a dietary-intake observational analysis.

## 5. Conclusions

Taken together, the present data provide no consistent evidence that habitual dietary omega-3 fatty acid exposure—whether captured at baseline or time-updated, and whether derived from marine or non-marine sources—meaningfully shapes the five-year trajectories of the five serum biomarkers of AF-related pathways examined in this Mediterranean cohort of older adults with metabolic syndrome. A modest cross-sectional direct association between marine omega-3 intake and 3-NT concentrations did not translate into longitudinal differences and should be interpreted cautiously, pending replication and a mechanistic study. Moderate non-marine omega-3 fatty acid intake at baseline showed an isolated inverse longitudinal association with PICP. Future analyses should evaluate the incidence of AF, stroke, and cardiovascular mortality.

## Figures and Tables

**Table 1:** Baseline characteristics of the participants of the PREDIMED-Plus study included in this analysis according to tertiles of omega-3 fatty acid intake.

Characteristic	Tertile 1 (<1.76 g/d) *n* = 170	Tertile 2 (1.76–2.37 g/d) *n* = 170	Tertile 3 (2.37–5.56 g/d) *n* = 170
Age (years)	65 (5)	65 (5)	66 (5)
Female sex, %	44.1	42.4	35.3
University education, %	82.4	80.6	77.1
Married, %	75.9	82.4	76.9
Single, %	5.9	3.5	7.7
Widower, %	12.4	8.8	9.5
Separated/Divorced, %	5.9	5.3	5.9
Currently smoking, %	13.6	8.9	7.6
Former smoker, %	46.2	52.1	52.4
Never smoker, %	40.2	39.1	40.0
Body mass index (kg/m^2^)	32.5 (3.3)	32.3 (3.4)	31.6 (3.0)
Weight (kg)	86.7 (12.1)	87.6 (14.3)	86.1 (11.9)
Waist circumference (cm)	106.3 (7.9)	106.1 (9.0)	105.2 (8.0)
Physical activity (METs min/wk)	2022 (1993)	2534 (2238)	3051 (2454)
Intervention group, %	51.2	52.9	48.8
Diabetes, %	31.2	28.2	27.1
Hypercholesterolemia, %	69.4	71.2	77.8
Hypertension, %	90.0	85.8	85.5
Previous history of cancer, %	4.7	7.6	10.0
Obstructive sleep apnea syndrome, %	12.9	18.8	16.5
eGFR (ml/min/1.73 m^2^)	92.1 (10.6)	90.4 (12.4)	89.0 (12.9)
Adherence to Mediterranean diet (0–17 score)	6.7 (2.9)	7.5 (2.6)	8.9 (2.9)
Total energy intake (kcal/d)	2437 (606)	2230 (567)	2443 (538)
Total omega-3 (g/d)	1.46 (0.22)	2.03 (0.17)	3.05 (0.56)
Marine omega-3 (g/d)	0.40 (0.16)	0.58 (0.20)	0.79 (0.41)
Non-marine omega-3 (g/d)	1.06 (0.23)	1.44 (0.24)	2.26 (0.66)
NT-pro-BNP (pg/mL)	84.12 (158.66)	79.01 (147.12)	69.14 (57.89)
Hs-TnT (ng/L)	9.21 (4.52)	9.22 (5.48)	9.55 (4.24)
CRP (mg/dL)	0.46 (0.96)	0.38 (0.50)	0.37 (0.56)
PICP (mg/mL)	97.36 (35.52)	98.10 (45.03)	95.93 (45.73)
3-NT (nM)	696 (753)	720 (570)	847 (725)

Mean (SD) for continuous variables; n (%) for categorical variables.

## Data Availability

Data collaboration for the PREDIMED-Plus study is guided by the Data Sharing and Management guide. We follow a controlled data collaboration model, using anonymized (de-identified) study data only, for collaborating with approved researchers. Requests are considered by the PREDIMED-Plus Steering Committee (predimed_plus_scommittee@googlegroups.com). Decisions on data access are based on the scientific legitimacy of the requester and of their institution, and on assurances on information security and governance.

## Supplementary Materials

The following supporting information can be downloaded at: https://www.mdpi.com/article/10.3390/nu18111669/s1, Supplemental Figure S1: cross-sectional association between tertiles of baseline marine omega-3 fatty acid intake and baseline biomarkers of atrial fibrillation related pathways; Supplemental Figure S2: cross-sectional association between tertiles of baseline non-marine omega-3 fatty acid intake and baseline biomarkers of atrial fibrillation related pathways; Supplemental Figure S3: association between baseline tertiles of marine omega-3 fatty acid intake and biomarkers of atrial fibrillation related pathways over follow-up; Supplemental Figure S4: association between baseline tertiles of non-marine omega-3 fatty acid intake and biomarkers of atrial fibrillation related pathways over follow-up; Supplemental Figure S5: association between updated marine omega-3 fatty acid intake and biomarkers of atrial fibrillation related pathways over follow-up; Supplemental Figure S6: association between updated non-marine omega-3 fatty acid intake and biomarkers of atrial fibrillation related pathways over follow-up; Supplemental Figure S7: restricted cubic spline analysis of the dose–response between baseline non-marine omega-3 fatty acid intake and the procollagen type I C-terminal propeptide (PICP) at baseline, at year 5, and over follow-up; Supplemental Table S1: baseline characteristics of the three-site biomarker substudy (*n* = 510) versus the parent PREDIMED-Plus cohort (*n* = 6874); Supplemental Table S2a: sensitivity analyses to assess potential overadjustment for total omega-3 fatty acid intake (cross-sectional association); Supplemental Table S2b: sensitivity analyses to assess potential overadjustment for total omega-3 fatty acid intake (longitudinal mixed models); Supplemental Table S2c: sensitivity analyses to assess potential overadjustment for marine omega-3 fatty acid intake (cross-sectional, association); Supplemental Table S2d: sensitivity analyses to assess potential overadjustment for marine omega-3 fatty acid intake (longitudinal mixed models); Supplemental Table S2e: sensitivity analyses to assess potential overadjustment for non-marine omega-3 fatty acid intake (cross-sectional association); Supplemental Table S2f: sensitivity analyses to assess potential overadjustment for non-marine omega-3 fatty acid intake (longitudinal mixed models); Supplemental Table S3: comparison of baseline characteristics of retained participants vs. participants lost to follow-up; Supplemental Table S4a: IPAW-weighted vs. unweighted longitudinal analysis for marine omega-3 fatty acid intake; Supplemental Table S4b: IPAW-weighted vs. unweighted longitudinal analysis for non-marine omega-3 fatty acid intake; Supplemental Table S4c: IPAW-weighted vs. unweighted longitudinal analysis for total omega-3 fatty acid intake.
