# Supplementary material for: Effect of Omega-3 Fatty Acid Intake on Circulating Biomarkers of Atrial Fibrillation-Related Pathways in the PREDIMED-Plus Study
Source: Nutrients. 2026 May 23;18(11):1669. doi: 10.3390/nu18111669 (PMC13257648; doi:10.3390/nu18111669)
Supplement: Supplementary file 1 [file nutrients-18-01669-s001.zip › nutrients-4275386-supplementary.pdf]

Supplemental Material

Supplemental Figure S1. Cross-sectional association between tertiles of baseline marine omega-3 fatty acid intake and baseline biomarkers of atrial fibrillation related pathways.

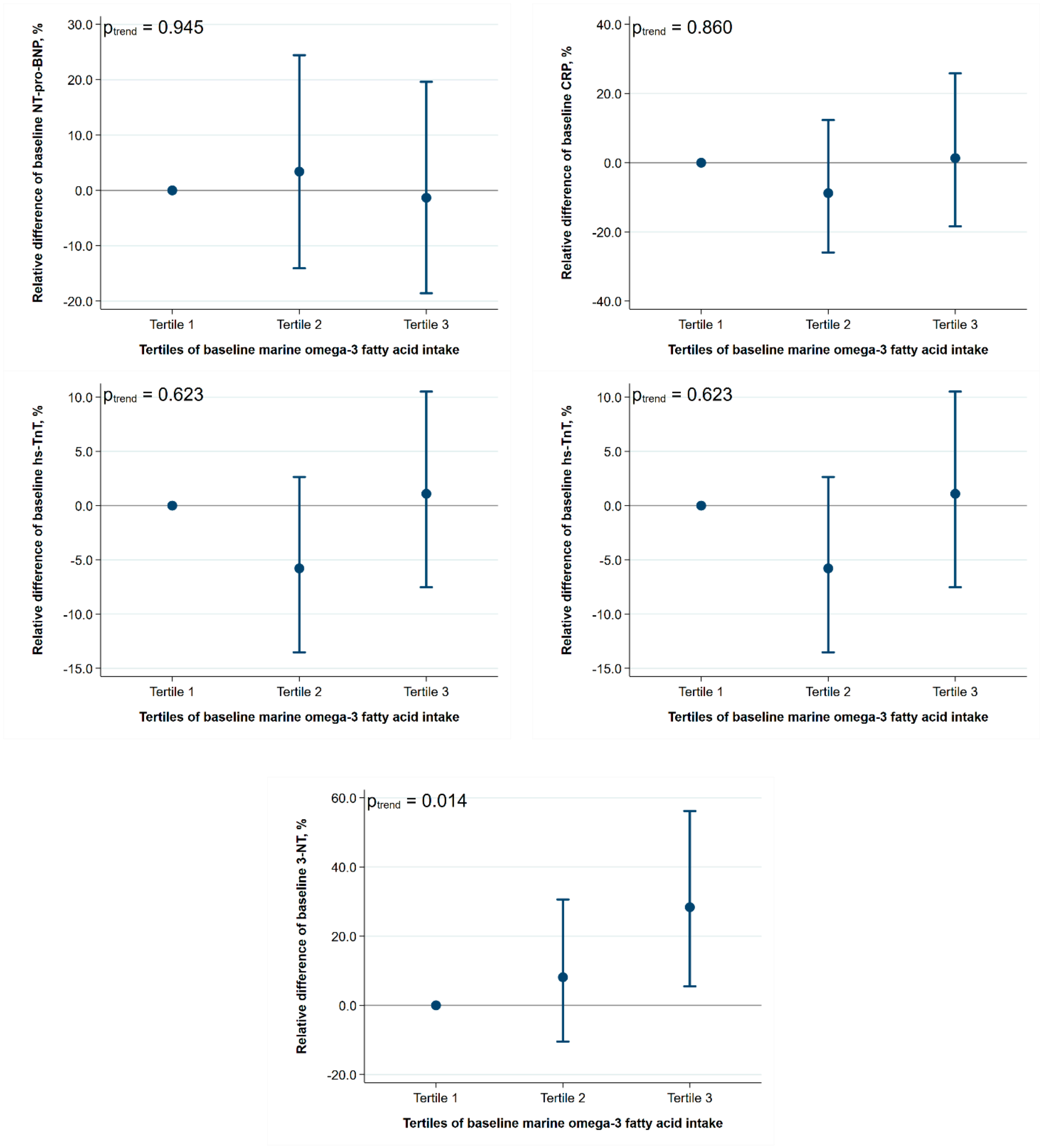

Multivariable adjusted model: Adjusted for sex, age, intervention group, recruitment center, prevalent diabetes, previous history of cancer, smoking habit at baseline (3 categories), civil status, prevalent dyslipidemia, prevalent hypertension, previous history of sleep apnea, eGFR, time of physical activity, BMI, total caloric intake, educational level, adherence to an energy-reduced Mediterranean diet and baseline tertiles of non-marine omega-3 intake.

Supplemental Figure S2. Cross-sectional association between tertiles of baseline non-marine omega-3 fatty acid intake and baseline biomarkers of atrial fibrillation related pathways.

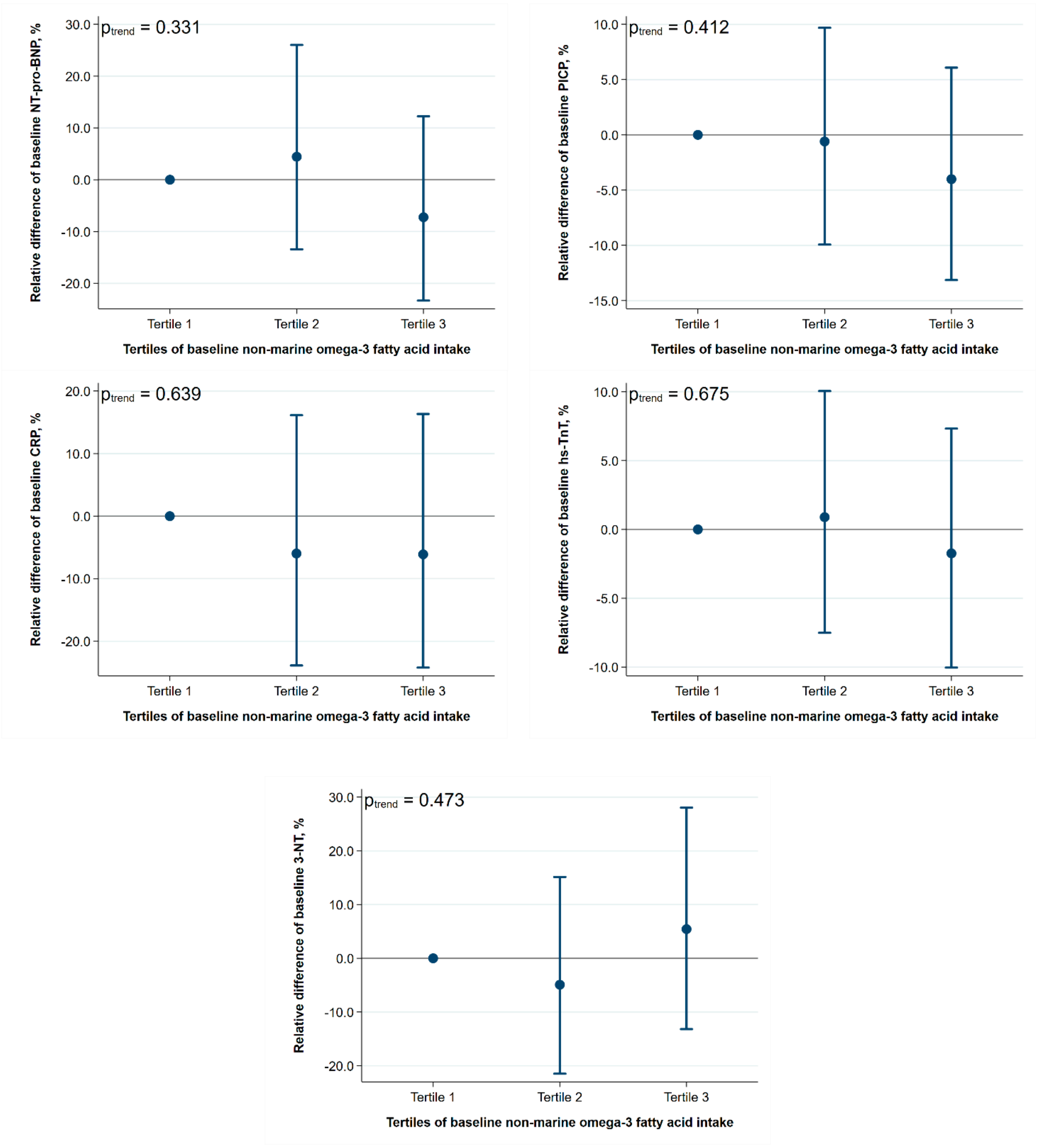

Multivariable adjusted model: Adjusted for sex, age, intervention group, recruitment center, prevalent diabetes, previous history of cancer, smoking habit at baseline (3 categories), civil status, prevalent dyslipidemia, prevalent hypertension, previous history of sleep apnea, eGFR, time of physical activity, BMI, total caloric intake, educational level, adherence to an energy-reduced Mediterranean diet and baseline tertiles of marine omega-3 intake.

Supplemental Figure S3. Association between baseline tertiles of marine omega-3 fatty acid intake and biomarkers of atrial fibrillation related pathways over follow-up.

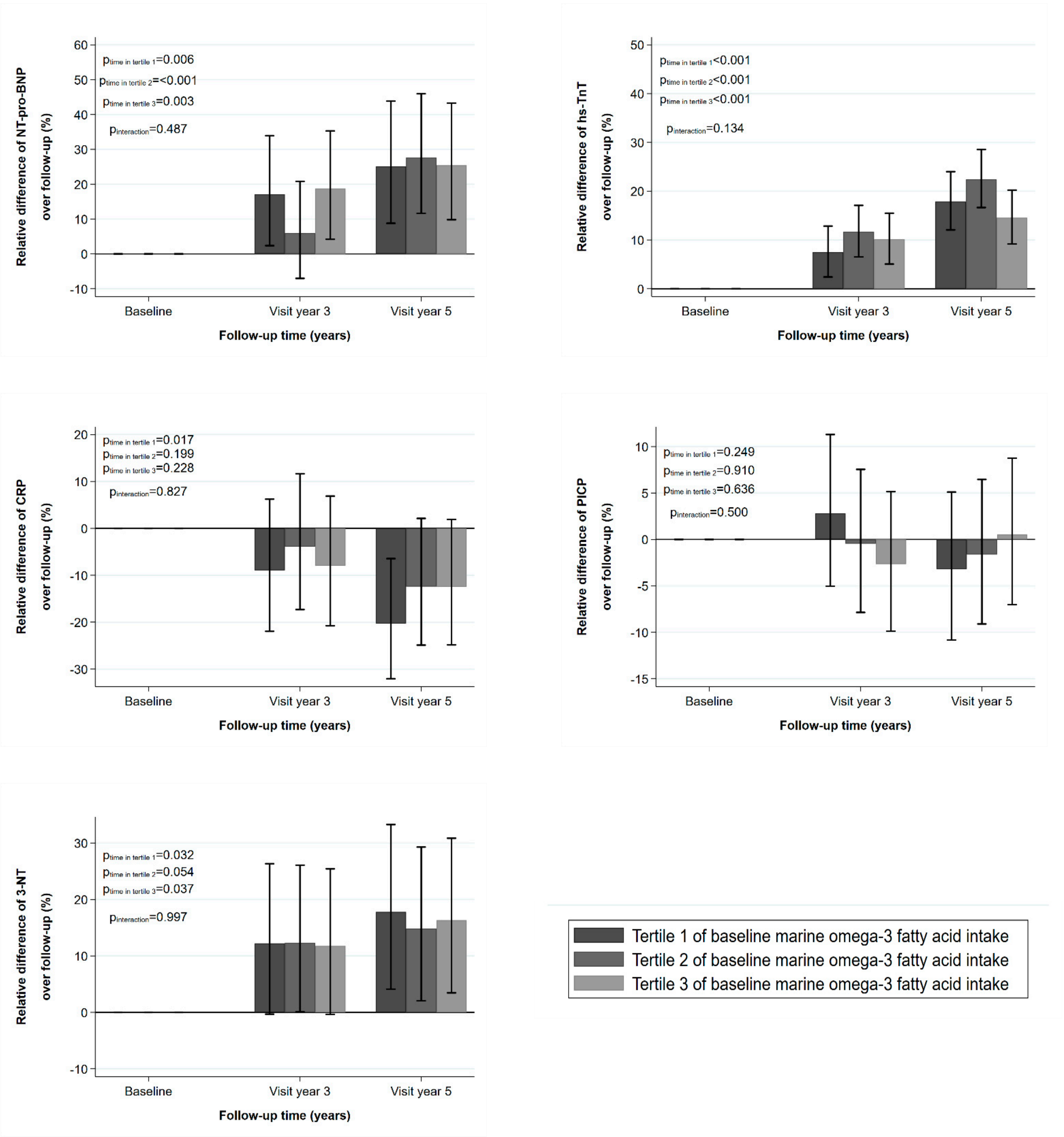

Multivariable adjusted model: Adjusted for sex, age, intervention group, recruitment center, prevalent diabetes, previous history of cancer, smoking habit at baseline (3 categories), civil status, prevalent dyslipidemia, prevalent hypertension, previous history of sleep apnea, eGFR, time of physical activity, BMI, total caloric intake, educational level, adherence to an energy-reduced Mediterranean diet and baseline tertiles of non-marine omega-3 intake.

Supplemental Figure S4. Association between baseline tertiles of non-marine omega-3 fatty acid intake and biomarkers of atrial fibrillation related pathways over follow-up.

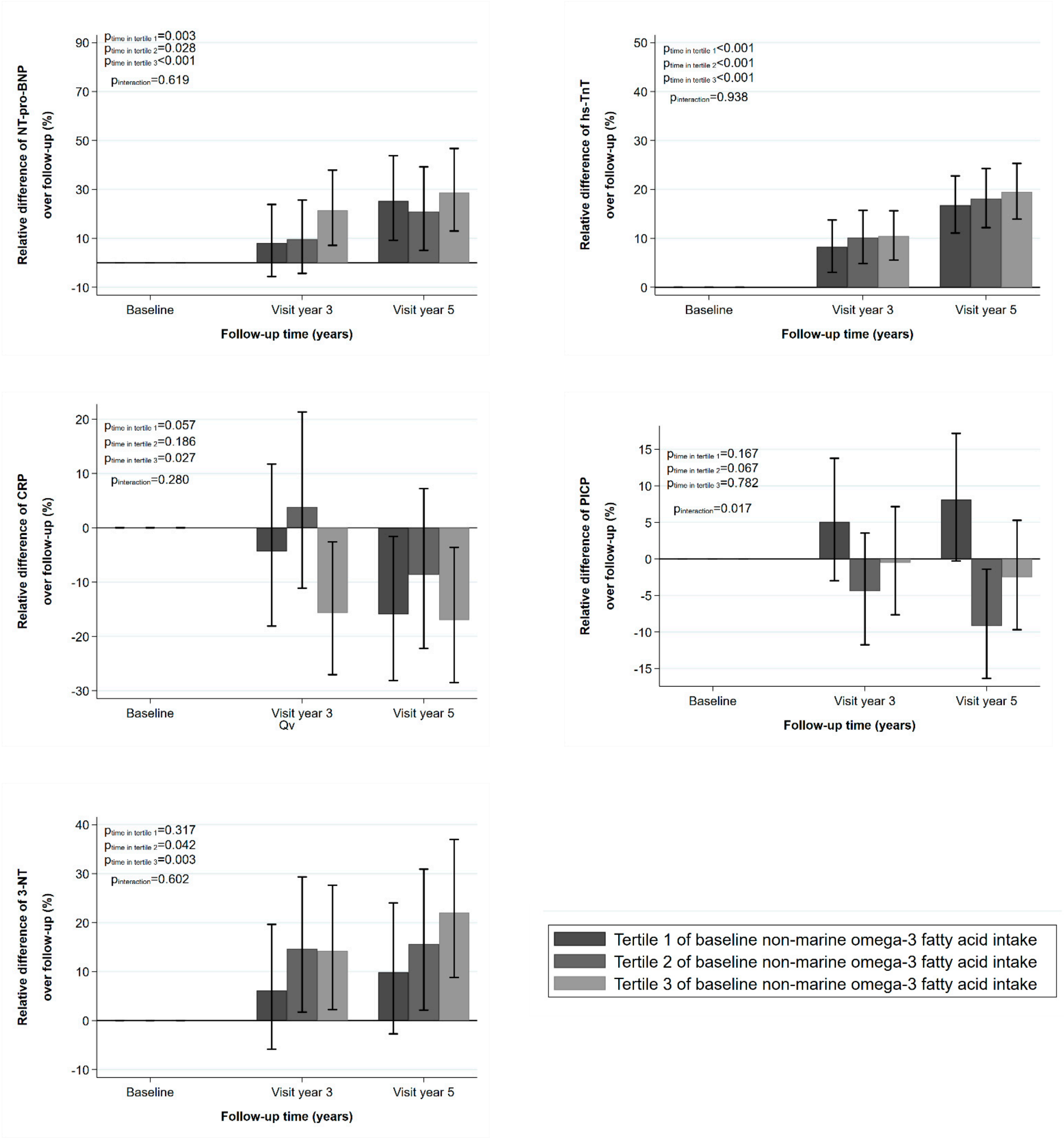

Multivariable adjusted model: Adjusted for sex, age, intervention group, recruitment center, prevalent diabetes, previous history of cancer, smoking habit at baseline (3 categories), civil status, prevalent dyslipidemia, prevalent hypertension, previous history of sleep apnea, eGFR, time of physical activity, BMI, total caloric intake, educational level, adherence to an energy-reduced Mediterranean diet and baseline tertiles of marine omega-3 intake.

Supplemental Figure S5. Association between updated marine omega-3 fatty acid intake and biomarkers of atrial fibrillation related pathways over follow-up.

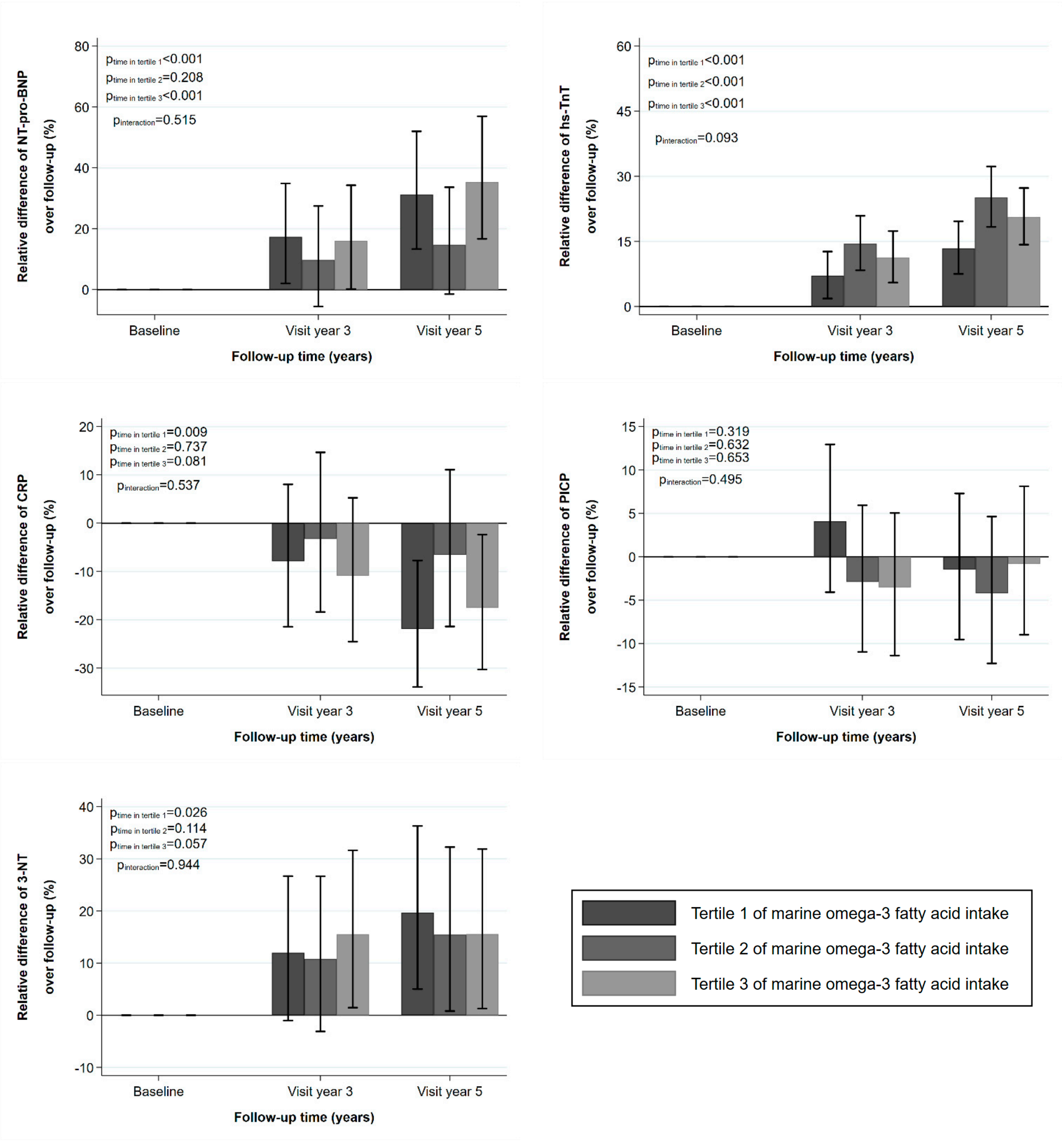

Multivariable adjusted model: Adjusted for sex, age, intervention group, recruitment center, prevalent diabetes, previous history of cancer, smoking habit at baseline (3 categories), civil status, prevalent dyslipidemia, prevalent hypertension, previous history of sleep apnea, eGFR, time of physical activity, BMI, total caloric intake, educational level, adherence to an energy-reduced Mediterranean diet and tertiles of non-marine omega intake at baseline.

Supplemental Figure S6. Association between updated non-marine omega-3 fatty acid intake and biomarkers of atrial fibrillation related pathways over follow-up.

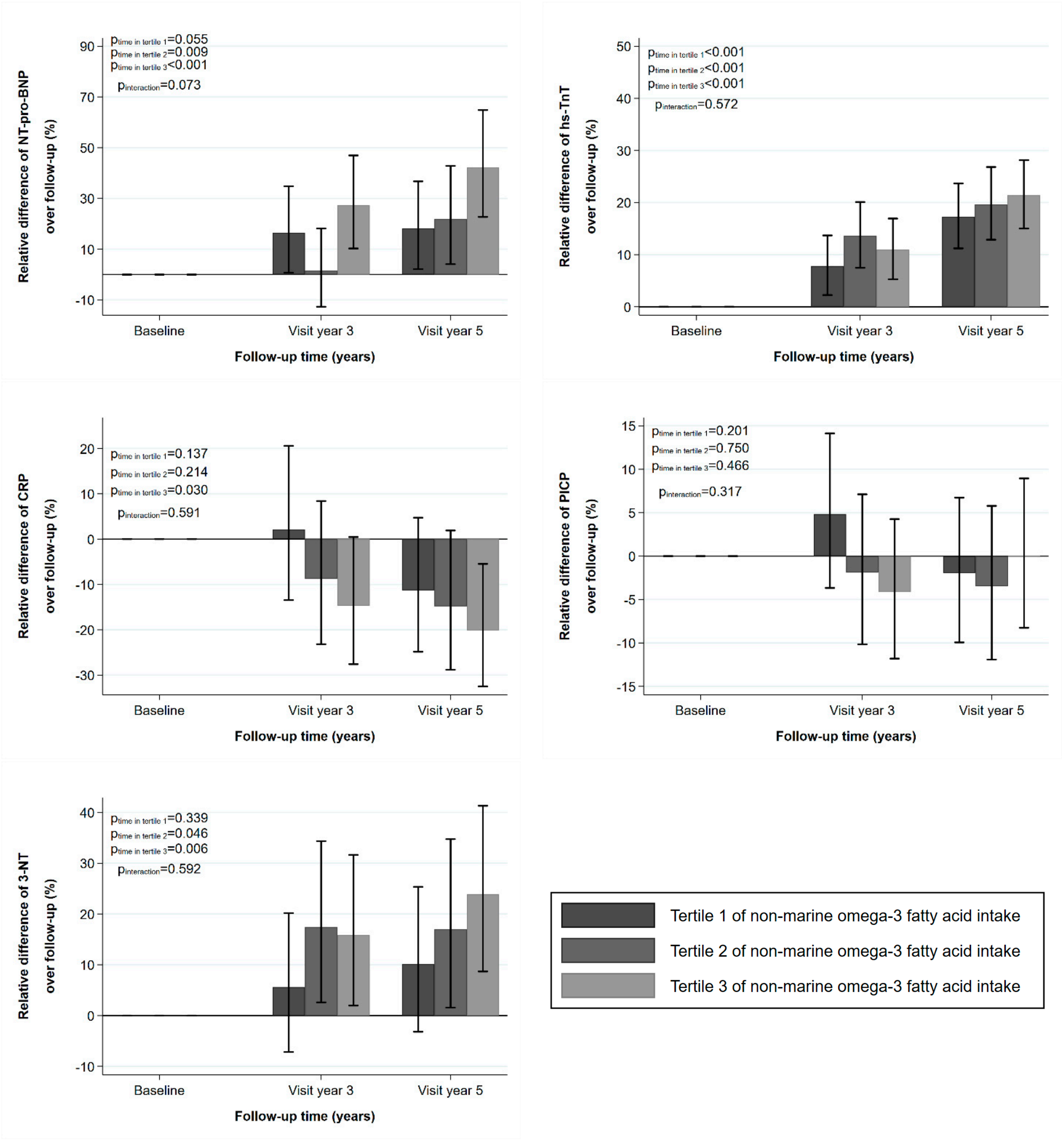

Multivariable adjusted model: Adjusted for sex, age, intervention group, recruitment center, prevalent diabetes, previous history of cancer, smoking habit at baseline (3 categories), civil status, prevalent dyslipidemia, prevalent hypertension, previous history of sleep apnea, eGFR, time of physical activity, BMI, total caloric intake, educational level, adherence to an energy-reduced Mediterranean diet and tertiles of marine omega intake at baseline.

Supplemental Figure S7. Restricted cubic spline analysis of the dose–response between baseline non-marine omega-3 fatty acid intake and the procollagen type I C-terminal propeptide (PICP) at baseline, at year 5, and over follow-up.

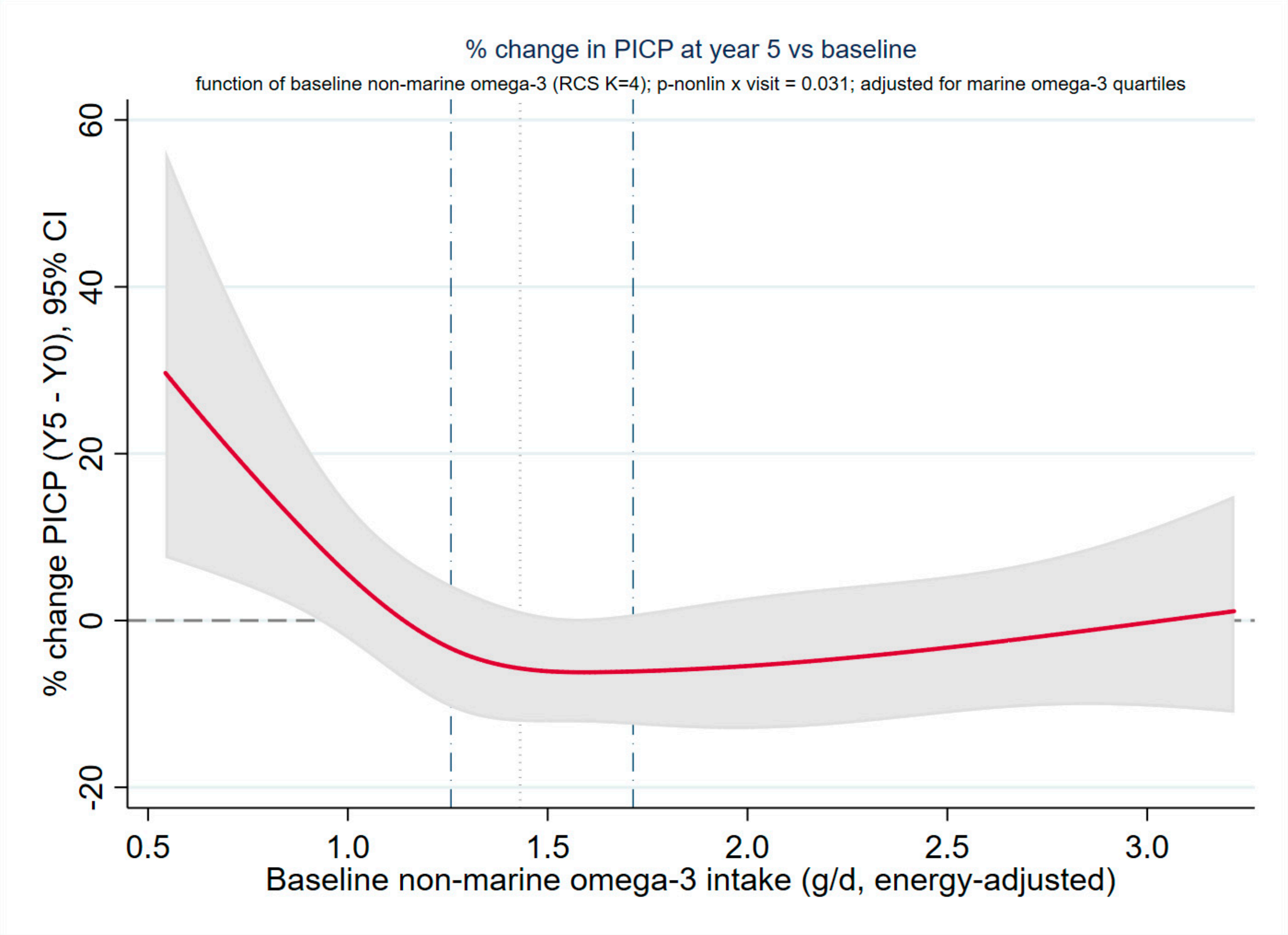

Restricted cubic splines for baseline non-marine omega-3 intake. Primary model: 4 knots at P5/P35/P65/P95; sensitivity: 3 and 5 knots. Models adjusted for total energy intake, sex, age, intervention group, prevalent diabetes, prevalent cancer, smoking status, marital status, prevalent dyslipidemia, prevalent hypertension, previous history of sleep apnea, total physical activity, BMI, adherence to an energy-reduced Mediterranean diet, eGFR, education level, recruitment center, and tertiles of baseline marine omega-3 (EPA+DHA+DHA) intake. Longitudinal model additionally includes random effects for cluster and subject. Vertical dash-dot lines in the longitudinal panel indicate baseline tertile cut-points; dotted line indicates the median of the exposure distribution used as reference.

p-nonlinearity:

- Cross-sectional baseline (visit 0): K=3 p=0.576; K=4 (primary) p=0.653; K=5 p=0.792.
- Cross-sectional year 5 (visit 5): K=3 p=0.005; K=4 (primary) p=0.018; K=5 p=0.018.
- Longitudinal mixed (RCS × visit): K=3 p=0.007; K=4 (primary) p=0.031; K=5 p=0.028.

Supplemental Table S1. Baseline characteristics of the three-site biomarker substudy (n=510) versus the parent PREDIMED-Plus cohort (n=6,874)

Variable	Substudy n=510	PREDIMED-Plus n=6,874	SMD / Δ%
Age (years)	65 (5)	65 (5)	0.06
Female sex, %	59.4%	48.5%	+10.9 %
University education, %	80.0%	77.7%	+2.3 %
Married, %	78.4%	76.5%	+1.9 %
Single (incl religious), %	5.7%	5.1%	+0.6 %
Widower, %	10.2%	10.4%	−0.2 %
Separated/Divorced, %	5.7%	7.9%	−2.2 %
Currently smoking, %	10.0%	12.5%	−2.5 %
Former smoker, %	50.2%	43.7%	+6.5 %
Never smoker, %	39.8%	43.9%	−4.1 %
Body-mass index (kg/m²)	32.1 (3.3)	32.6 (3.5)	-0.14
Weight (kg)	86.8 (12.8)	86.6 (13.0)	0.02
Waist circumference (cm)	105.9 (8.3)	107.6 (9.7)	-0.19
Physical activity (METs-min/wk)	2536 (2271)	2462 (2300)	0.03
Intervention group, %	51.0%	50.4%	+0.6 %
Diabetes, %	28.8%	30.9%	−2.1 %
Hypercholesterolemia, %	72.9%	70.0%	+2.9 %
Hypertension, %	87.1%	83.8%	+3.3 %
Previous history of cancer, %	7.5%	7.2%	+0.3 %
Obstructive sleep apnea syndrome, %	16.1%	12.9%	+3.2 %
eGFR (ml/min/1.73 m^2)	90.5 (12.0)	88.5 (14.0)	0.16
Adherence to Mediterranean Diet (0-17 score)	7.7 (2.9)	8.5 (2.7)	-0.28
Total energy intake (kcal/d)	2370 (578)	2416 (633)	-0.08
Total omega-3 (g/d)	2.17 (0.75)	2.39 (0.95)	-0.23
Marine omega-3 (g/d)	0.59 (0.32)	0.74 (0.41)	-0.47
Non-marine omega-3 (g/d)	1.58 (0.66)	1.64 (0.75)	-0.05
NT-pro-BNP (pg/mL)	77.43 (129.21)	—	—
Hs-TnT (ng/L)	9.33 (4.77)	—	—
CRP (mg/dL)	0.40 (0.70)	—	—
PICP (mg/mL)	97.13 (42.28)	—	—
3-NT (nM)	754 (689)	—	—

Continuous variables: mean (SD). Binary variables: proportions. Both columns were computed from individual-level baseline data: the substudy column from the analytic dataset of the present study after the pre-specified exclusions (implausible energy intake, prior atrial fibrillation, and absence of any baseline biomarker measurement); the parent-cohort column from the full PREDIMED-Plus baseline dataset (n=6,874).

SMD continuous = (mean in substudy − mean in overall cohort) / pooled SD.

Δ% = % in substudy − % in overall cohort.

'—': cohort-level value not available for biomarkers.

Supplemental Table S2a. Sensitivity analyses to assess potential overadjustment for total omega-3 fatty acid intake (cross-sectional association)

Biomarker	Specification	T2 vs T1: %diff (95% CI)	p (T2)	T3 vs T1: %diff (95% CI)	p (T3)	p-trend (continuous)
NT-pro-BNP	Fully adjusted	-4.0 (-20.0, 15.1)	0.659	-5.1 (-21.7, 14.9)	0.590	0.617
NT-pro-BNP	W/o BMI	-4.0 (-19.9, 15.2)	0.662	-4.6 (-21.2, 15.6)	0.633	0.665
NT-pro-BNP	W/o erMedDiet	-4.1 (-20.0, 15.0)	0.654	-5.5 (-21.6, 13.8)	0.549	0.577
NT-pro-BNP	W/o BMI and erMedDiet	-4.0 (-20.0, 15.1)	0.658	-4.9 (-21.0, 14.5)	0.599	0.632
hs-TnT	Fully adjusted	-3.0 (-10.8, 5.6)	0.482	-2.0 (-10.3, 7.2)	0.662	0.734
hs-TnT	W/o BMI	-3.0 (-10.9, 5.6)	0.478	-2.6 (-10.9, 6.4)	0.557	0.617
hs-TnT	W/o erMedDiet	-2.8 (-10.7, 5.7)	0.502	-0.9 (-9.1, 8.0)	0.831	0.928
hs-TnT	W/o BMI and erMedDiet	-2.9 (-10.8, 5.7)	0.495	-1.7 (-9.9, 7.2)	0.697	0.780
CRP	Fully adjusted	-10.8 (-27.3, 9.4)	0.272	-7.9 (-25.7, 14.3)	0.456	0.549
CRP	W/o BMI	-11.0 (-27.7, 9.6)	0.273	-10.7 (-28.3, 11.3)	0.314	0.379
CRP	W/o erMedDiet	-11.1 (-27.5, 9.1)	0.259	-10.1 (-27.1, 10.8)	0.319	0.397
CRP	W/o BMI and erMedDiet	-11.3 (-28.0, 9.2)	0.258	-13.2 (-29.9, 7.4)	0.191	0.237
PICP	Fully adjusted	-1.8 (-10.8, 8.0)	0.704	-4.4 (-13.6, 5.7)	0.379	0.379
PICP	W/o BMI	-1.8 (-10.8, 8.0)	0.701	-4.7 (-13.8, 5.4)	0.349	0.348
PICP	W/o erMedDiet	-1.8 (-10.7, 8.1)	0.714	-3.9 (-12.8, 6.0)	0.424	0.427
PICP	W/o BMI and erMedDiet	-1.8 (-10.7, 8.0)	0.711	-4.2 (-13.1, 5.6)	0.388	0.388
3-NT	Fully adjusted	3.1 (-14.4, 24.2)	0.746	16.3 (-4.4, 41.6)	0.130	0.116
3-NT	W/o BMI	3.2 (-14.4, 24.4)	0.741	17.6 (-3.3, 43.2)	0.105	0.091
3-NT	W/o erMedDiet	2.7 (-14.8, 23.7)	0.781	12.5 (-7.1, 36.1)	0.227	0.208
3-NT	W/o BMI and erMedDiet	2.8 (-14.7, 23.9)	0.774	13.9 (-5.9, 37.8)	0.182	0.163

Percent difference (95% CI) in ln(biomarker) across tertiles of total omega-3 fatty acid intake. T1 = reference.

Fully adjusted model adjusted for total energy intake (kcal/day), sex, age (years), intervention group, prevalent type 2 diabetes, prevalent cancer, smoking status (never / former / current), marital status, hypercholesterolemia, hypertension, sleep apnea, leisure-time physical activity (METs·min/week), body-mass index (kg/m²), 17-item erMedDiet adherence score, estimated glomerular filtration rate (mL/min/1.73 m²) and educational level.

Sensitivity specifications were identical to the fully adjusted model except as follows:

- **No-BMI model**: body-mass index removed.
- **No-erMedDiet model**: 17-item erMedDiet adherence score removed.
- **No-both model**: both body-mass index and the 17-item erMedDiet adherence score removed.

3-NT, 3-nitrotyrosine; BMI, body-mass index; CI, confidence interval; CRP, C-reactive protein (high-sensitivity); eGFR, estimated glomerular filtration rate; erMedDiet, adherence to the energy-reduced Mediterranean diet; hs-TnT, high-sensitivity cardiac troponin T; METs, metabolic equivalents of task; NT-pro-BNP, N-terminal pro–B-type natriuretic peptide; PICP, procollagen type I C-terminal propeptide; T1/T2/T3, tertile 1 / tertile 2 / tertile 3 of exposure (T1 = reference); W/o, without.

Supplemental Table S2b. Sensitivity analyses to assess potential overadjustment for total omega-3 fatty acid intake (longitudinal mixed models)

Biomarker	Specification	T1: Y3-Y0 %diff (95% CI)	T1: Y5-Y0 %diff (95% CI)	T2: Y3-Y0 %diff (95% CI)	T2: Y5-Y0 %diff (95% CI)	T3: Y3-Y0 %diff (95% CI)	T3: Y5-Y0 %diff (95% CI)	p-interaction (tertile#visit, Wald)
NT-pro-BNP	Fully adjusted	8.2 (-5.5, 23.9)	24.1 (8.2, 42.3)	13.4 (-1.0, 30.0)	21.0 (4.8, 39.7)	17.4 (3.5, 33.3)	30.0 (14.2, 47.9)	0.812
NT-pro-BNP	W/o BMI	8.2 (-5.5, 23.9)	24.1 (8.2, 42.4)	13.4 (-1.0, 30.0)	21.0 (4.8, 39.7)	17.5 (3.5, 33.3)	30.0 (14.2, 47.9)	0.812
NT-pro-BNP	W/o erMedDiet	14.1 (1.5, 28.2)	30.5 (15.7, 47.1)	19.5 (6.3, 34.4)	28.1 (13.5, 44.7)	21.0 (7.6, 36.2)	34.5 (19.2, 51.8)	0.911
NT-pro-BNP	W/o BMI and erMedDiet	14.1 (1.5, 28.2)	30.5 (15.7, 47.1)	19.5 (6.3, 34.4)	28.1 (13.5, 44.7)	21.0 (7.6, 36.2)	34.5 (19.2, 51.8)	0.911
hs-TnT	Fully adjusted	8.3 (3.1, 13.8)	16.0 (10.4, 21.9)	9.1 (3.8, 14.6)	19.2 (13.2, 25.7)	11.4 (6.4, 16.6)	19.0 (13.6, 24.7)	0.786
hs-TnT	W/o BMI	8.4 (3.2, 13.9)	16.1 (10.5, 22.0)	9.2 (3.9, 14.7)	19.4 (13.3, 25.8)	11.4 (6.4, 16.6)	19.1 (13.6, 24.8)	0.792
hs-TnT	W/o erMedDiet	8.9 (4.4, 13.6)	16.7 (11.7, 21.8)	9.7 (5.2, 14.4)	20.0 (14.9, 25.4)	11.9 (7.2, 16.7)	19.5 (14.4, 24.8)	0.788
hs-TnT	W/o BMI and erMedDiet	9.0 (4.5, 13.6)	16.7 (11.8, 21.9)	9.8 (5.3, 14.5)	20.1 (14.9, 25.5)	11.9 (7.2, 16.7)	19.5 (14.4, 24.8)	0.793
CRP	Fully adjusted	-6.5 (-20.0, 9.1)	-17.5 (-29.4, -3.5)	4.4 (-10.6, 22.0)	-6.7 (-20.8, 9.9)	-14.9 (-26.3, -1.6)	-17.6 (-28.9, -4.6)	0.284
CRP	W/o BMI	-5.7 (-19.2, 10.1)	-16.6 (-28.7, -2.5)	5.4 (-9.8, 23.1)	-5.8 (-20.0, 11.0)	-14.4 (-26.0, -1.1)	-17.3 (-28.6, -4.1)	0.266
CRP	W/o erMedDiet	-7.9 (-19.4, 5.3)	-18.5 (-28.9, -6.4)	3.1 (-9.9, 17.9)	-8.0 (-20.0, 5.8)	-15.3 (-26.0, -3.0)	-18.3 (-28.9, -6.3)	0.310
CRP	W/o BMI and erMedDiet	-7.4 (-19.0, 5.8)	-18.0 (-28.5, -5.9)	3.6 (-9.4, 18.5)	-7.5 (-19.6, 6.3)	-15.1 (-25.8, -2.8)	-18.2 (-28.7, -6.1)	0.298
PICP	Fully adjusted	2.0 (-5.9, 10.5)	1.3 (-6.5, 9.9)	0.1 (-7.6, 8.5)	-4.4 (-12.2, 4.0)	-1.7 (-8.8, 6.0)	-0.5 (-7.8, 7.4)	0.705
PICP	W/o BMI	1.9 (-5.9, 10.4)	1.3 (-6.5, 9.8)	0.1 (-7.7, 8.4)	-4.5 (-12.2, 3.9)	-1.7 (-8.8, 5.9)	-0.5 (-7.8, 7.4)	0.705
PICP	W/o erMedDiet	1.1 (-5.7, 8.4)	0.5 (-6.4, 7.9)	-0.8 (-7.5, 6.4)	-5.4 (-11.9, 1.7)	-2.3 (-8.9, 4.8)	-1.0 (-7.9, 6.3)	0.692
PICP	W/o BMI and erMedDiet	1.1 (-5.7, 8.3)	0.5 (-6.4, 7.9)	-0.8 (-7.5, 6.3)	-5.4 (-11.9, 1.7)	-2.3 (-8.9, 4.8)	-1.0 (-7.9, 6.3)	0.692
3-NT	Fully adjusted	7.5 (-4.6, 21.2)	8.6 (-3.8, 22.5)	18.8 (5.4, 34.0)	24.1 (9.3, 40.9)	9.9 (-1.7, 22.8)	16.7 (4.1, 30.7)	0.423
3-NT	W/o BMI	7.4 (-4.7, 21.1)	8.5 (-3.9, 22.4)	18.7 (5.3, 33.8)	23.9 (9.2, 40.7)	9.8 (-1.7, 22.7)	16.6 (4.1, 30.7)	0.424
3-NT	W/o erMedDiet	2.6 (-7.4, 13.6)	3.7 (-6.7, 15.1)	13.2 (2.2, 25.5)	17.7 (5.8, 30.9)	6.5 (-4.0, 18.1)	13.0 (1.6, 25.6)	0.459
3-NT	W/o BMI and erMedDiet	2.5 (-7.4, 13.5)	3.6 (-6.7, 15.1)	13.2 (2.1, 25.4)	17.6 (5.7, 30.8)	6.5 (-4.0, 18.0)	13.0 (1.6, 25.6)	0.459

Percent change (95% CI) in ln(biomarker) at year 3 and year 5 within each tertile of total omega-3 fatty acid intake (vs baseline).

Linear mixed models with random intercepts were used

Fully adjusted model adjusted for total energy intake (kcal/day), sex, age (years), intervention group, prevalent type 2 diabetes, prevalent cancer, smoking status (never / former / current), marital status, hypercholesterolemia, hypertension, sleep apnea, leisure-time physical activity (METs·min/week), body-mass index (kg/m²), 17-item erMedDiet adherence score, estimated glomerular filtration rate (mL/min/1.73 m²) and educational level.

Sensitivity specifications were identical to the fully adjusted model except as follows: **No-BMI model**: body-mass index removed. **No-erMedDiet model**: 17-item erMedDiet adherence score removed.**No-both model**: both body-mass index and the 17-item erMedDiet adherence score removed.

3-NT, 3-nitrotyrosine; BMI, body-mass index; CI, confidence interval; CRP, C-reactive protein (high-sensitivity); eGFR, estimated glomerular filtration rate; erMedDiet, adherence to the energy-reduced Mediterranean diet; hs-TnT, high-sensitivity cardiac troponin T; METs, metabolic equivalents of task; NT-pro-BNP, N-terminal pro–B-type natriuretic peptide; PICP, procollagen type I C-terminal propeptide; T1/T2/T3, tertile 1 / tertile 2 / tertile 3 of exposure (T1 = reference); W/o, without.

Supplemental Table S2c. Sensitivity analyses to assess potential overadjustment for marine omega-3 fatty acid intake (cross-sectional association)

Biomarker	Specification	T2 vs T1: %diff (95% CI)	p (T2)	T3 vs T1: %diff (95% CI)	p (T3)	p-trend (continuous)
NT-pro-BNP	Fully adjusted	3.4 (-14.0, 24.4)	0.723	-1.3 (-18.6, 19.6)	0.891	0.945
NT-pro-BNP	W/o BMI	3.4 (-14.0, 24.4)	0.721	-1.2 (-18.5, 19.7)	0.901	0.957
NT-pro-BNP	W/o erMedDiet	3.3 (-13.8, 23.9)	0.722	-1.4 (-18.1, 18.6)	0.880	0.921
NT-pro-BNP	W/o BMI and erMedDiet	3.5 (-13.7, 24.0)	0.713	-1.2 (-17.9, 18.9)	0.900	0.943
hs-TnT	Fully adjusted	-5.8 (-13.5, 2.6)	0.171	1.1 (-7.5, 10.5)	0.812	0.623
hs-TnT	W/o BMI	-5.8 (-13.6, 2.6)	0.172	1.0 (-7.7, 10.4)	0.835	0.651
hs-TnT	W/o erMedDiet	-5.0 (-12.6, 3.4)	0.236	2.4 (-6.1, 11.5)	0.594	0.474
hs-TnT	W/o BMI and erMedDiet	-5.1 (-12.7, 3.3)	0.227	2.1 (-6.3, 11.3)	0.638	0.517
CRP	Fully adjusted	-8.8 (-25.9, 12.3)	0.385	1.3 (-18.4, 25.8)	0.905	0.860
CRP	W/o BMI	-8.9 (-26.3, 12.6)	0.389	0.7 (-19.2, 25.6)	0.948	0.914
CRP	W/o erMedDiet	-10.5 (-27.0, 9.7)	0.285	-1.4 (-19.9, 21.5)	0.895	0.953
CRP	W/o BMI and erMedDiet	-11.0 (-27.7, 9.6)	0.271	-2.6 (-21.2, 20.5)	0.809	0.860
PICP	Fully adjusted	-6.5 (-15.2, 3.0)	0.173	-4.7 (-13.8, 5.4)	0.353	0.427
PICP	W/o BMI	-6.5 (-15.2, 3.0)	0.172	-4.7 (-13.8, 5.4)	0.348	0.420
PICP	W/o erMedDiet	-5.9 (-14.5, 3.4)	0.207	-3.8 (-12.7, 6.0)	0.434	0.493
PICP	W/o BMI and erMedDiet	-6.0 (-14.5, 3.4)	0.204	-3.9 (-12.8, 5.9)	0.421	0.479
3-NT	Fully adjusted	8.1 (-10.4, 30.5)	0.416	28.4 (5.5, 56.1)	0.012	0.014
3-NT	W/o BMI	8.2 (-10.4, 30.7)	0.415	28.6 (5.7, 56.5)	0.012	0.013
3-NT	W/o erMedDiet	4.6 (-13.1, 25.9)	0.632	22.5 (1.4, 48.1)	0.036	0.035
3-NT	W/o BMI and erMedDiet	4.8 (-12.9, 26.2)	0.619	23.0 (1.8, 48.7)	0.032	0.031

Percent change (95% CI) in ln(biomarker) across tertiles of marine omega-3 fatty acid intake. T1 = reference.

Fully adjusted model adjusted for total energy intake (kcal/day), sex, age (years), intervention group, prevalent type 2 diabetes, prevalent cancer, smoking status (never / former / current), marital status, hypercholesterolemia, hypertension, sleep apnea, leisure-time physical activity (METs·min/week), body-mass index (kg/m²), 17-item erMedDiet adherence score, estimated glomerular filtration rate (mL/min/1.73 m²) and educational level.

Sensitivity specifications were identical to the fully adjusted model except as follows:

- **No-BMI model**: body-mass index removed.
- **No-erMedDiet model**: 17-item erMedDiet adherence score removed.
- **No-both model**: both body-mass index and the 17-item erMedDiet adherence score removed.

3-NT, 3-nitrotyrosine; BMI, body-mass index; CI, confidence interval; CRP, C-reactive protein (high-sensitivity); eGFR, estimated glomerular filtration rate; erMedDiet, adherence to the energy-reduced Mediterranean diet; hs-TnT, high-sensitivity cardiac troponin T; METs, metabolic equivalents of task; NT-pro-BNP, N-terminal pro–B-type natriuretic peptide; PICP, procollagen type I C-terminal propeptide; T1/T2/T3, tertile 1 / tertile 2 / tertile 3 of exposure (T1 = reference); W/o, without.

Supplemental Table S2d. Sensitivity analyses to assess potential overadjustment for basal marine omega-3 fatty acid intake (longitudinal mixed models)

Biomarker	Specification	T1: Y3-Y0 %diff (95% CI)	T1: Y5-Y0 %diff (95% CI)	T2: Y3-Y0 %diff (95% CI)	T2: Y5-Y0 %diff (95% CI)	T3: Y3-Y0 %diff (95% CI)	T3: Y5-Y0 %diff (95% CI)	p-interaction (tertile#visit, Wald)
NT-pro-BNP	Fully adjusted	17.1 (2.4, 33.9)	25.1 (8.8, 43.9)	6.0 (-7.0, 20.8)	27.6 (11.6, 45.9)	18.8 (4.2, 35.3)	25.4 (9.8, 43.3)	0.487
NT-pro-BNP	W/o BMI	17.1 (2.4, 33.9)	25.1 (8.8, 43.9)	6.0 (-7.0, 20.8)	27.7 (11.6, 46.0)	18.8 (4.2, 35.3)	25.4 (9.8, 43.3)	0.487
NT-pro-BNP	W/o erMedDiet	22.4 (8.9, 37.5)	31.0 (16.1, 47.7)	9.8 (-2.2, 23.4)	32.8 (17.8, 49.6)	23.0 (9.3, 38.5)	30.0 (15.2, 46.7)	0.454
NT-pro-BNP	W/o BMI and erMedDiet	22.4 (8.9, 37.5)	31.0 (16.1, 47.7)	9.8 (-2.2, 23.4)	32.8 (17.8, 49.6)	23.0 (9.3, 38.5)	30.0 (15.2, 46.7)	0.454
hs-TnT	Fully adjusted	7.5 (2.4, 12.8)	17.9 (12.1, 24.0)	11.7 (6.5, 17.1)	22.4 (16.6, 28.5)	10.2 (5.1, 15.5)	14.6 (9.2, 20.2)	0.134
hs-TnT	W/o BMI	7.6 (2.5, 12.9)	18.0 (12.2, 24.1)	11.8 (6.6, 17.2)	22.5 (16.7, 28.6)	10.2 (5.1, 15.6)	14.6 (9.2, 20.3)	0.133
hs-TnT	W/o erMedDiet	7.9 (3.5, 12.5)	18.4 (13.4, 23.7)	12.2 (7.6, 17.0)	22.9 (17.7, 28.3)	10.5 (5.9, 15.3)	15.0 (10.1, 20.1)	0.129
hs-TnT	W/o BMI and erMedDiet	7.9 (3.5, 12.5)	18.5 (13.5, 23.7)	12.2 (7.7, 17.0)	23.0 (17.8, 28.4)	10.6 (6.0, 15.4)	15.0 (10.1, 20.1)	0.128
CRP	Fully adjusted	-9.0 (-22.0, 6.2)	-20.3 (-32.0, -6.4)	-3.9 (-17.3, 11.6)	-12.4 (-24.9, 2.1)	-8.0 (-20.8, 6.9)	-12.5 (-24.8, 1.9)	0.827
CRP	W/o BMI	-8.2 (-21.3, 7.1)	-19.5 (-31.4, -5.5)	-3.2 (-16.7, 12.5)	-11.8 (-24.3, 2.9)	-7.4 (-20.3, 7.5)	-12.0 (-24.5, 2.4)	0.842
CRP	W/o erMedDiet	-9.1 (-20.5, 4.0)	-20.2 (-30.5, -8.3)	-3.6 (-15.7, 10.2)	-12.4 (-23.6, 0.5)	-7.9 (-19.6, 5.5)	-12.4 (-23.8, 0.6)	0.823
CRP	W/o BMI and erMedDiet	-8.6 (-20.1, 4.5)	-19.7 (-30.1, -7.8)	-3.1 (-15.3, 10.7)	-12.0 (-23.3, 1.0)	-7.6 (-19.4, 5.8)	-12.2 (-23.6, 0.8)	0.835
PICP	Fully adjusted	2.8 (-5.0, 11.3)	-3.2 (-10.8, 5.1)	-0.5 (-7.9, 7.6)	-1.6 (-9.1, 6.5)	-2.7 (-9.9, 5.1)	0.6 (-7.0, 8.8)	0.500
PICP	W/o BMI	2.8 (-5.1, 11.3)	-3.2 (-10.9, 5.1)	-0.5 (-7.9, 7.5)	-1.7 (-9.1, 6.4)	-2.7 (-9.9, 5.1)	0.5 (-7.0, 8.7)	0.499
PICP	W/o erMedDiet	2.1 (-4.8, 9.4)	-3.9 (-10.6, 3.2)	-1.2 (-7.8, 5.9)	-2.3 (-9.0, 4.9)	-3.2 (-9.8, 3.8)	-0.0 (-7.0, 7.4)	0.494
PICP	W/o BMI and erMedDiet	2.0 (-4.8, 9.3)	-4.0 (-10.6, 3.1)	-1.2 (-7.8, 5.9)	-2.3 (-9.0, 4.9)	-3.3 (-9.8, 3.8)	-0.1 (-7.0, 7.4)	0.493
3-NT	Fully adjusted	12.2 (-0.3, 26.3)	17.8 (4.1, 33.3)	12.3 (0.1, 26.1)	14.9 (2.1, 29.3)	11.8 (-0.4, 25.4)	16.4 (3.5, 30.9)	0.997
3-NT	W/o BMI	12.1 (-0.4, 26.2)	17.7 (4.0, 33.1)	12.2 (-0.0, 26.0)	14.8 (2.0, 29.2)	11.7 (-0.4, 25.3)	16.3 (3.4, 30.8)	0.997
3-NT	W/o erMedDiet	7.1 (-3.3, 18.6)	12.1 (0.8, 24.6)	7.7 (-2.7, 19.3)	10.1 (-0.9, 22.3)	7.6 (-3.1, 19.4)	11.9 (0.6, 24.5)	0.998
3-NT	W/o BMI and erMedDiet	7.0 (-3.3, 18.5)	12.0 (0.8, 24.5)	7.7 (-2.8, 19.2)	10.1 (-0.9, 22.2)	7.5 (-3.1, 19.3)	11.9 (0.6, 24.5)	0.998

Percent change (95% CI) in ln(biomarker) at year 3 and year 5 within each tertile of marine omega-3 fatty acid intake (vs baseline).

Linear mixed models with random intercepts were used

Fully adjusted model adjusted for total energy intake (kcal/day), sex, age (years), intervention group, prevalent type 2 diabetes, prevalent cancer, smoking status (never / former / current), marital status, hypercholesterolemia, hypertension, sleep apnea, leisure-time physical activity (METs·min/week), body-mass index (kg/m²), 17-item erMedDiet adherence score, estimated glomerular filtration rate (mL/min/1.73 m²) and educational level.

Sensitivity specifications were identical to the fully adjusted model except as follows: **No-BMI model**: body-mass index removed. **No-erMedDiet model**: 17-item erMedDiet adherence score removed. **No-both model**: both body-mass index and the 17-item erMedDiet adherence score removed.

3-NT, 3-nitrotyrosine; BMI, body-mass index; CI, confidence interval; CRP, C-reactive protein (high-sensitivity); eGFR, estimated glomerular filtration rate; erMedDiet, adherence to the energy-reduced Mediterranean diet; hs-TnT, high-sensitivity cardiac troponin T; METs, metabolic equivalents of task; NT-pro-BNP, N-terminal pro–B-type natriuretic peptide; PICP, procollagen type I C-terminal propeptide; T1/T2/T3, tertile 1 / tertile 2 / tertile 3 of exposure (T1 = reference); W/o, without.

Supplemental Table S2e. Sensitivity analyses to assess potential overadjustment for non-marine omega-3 fatty acid intake (cross-sectional association)

**Biomarker**	**Specification**	**T2 vs T1: %diff (95% CI)**	**p (T2)**	**T3 vs T1: %diff (95% CI)**	**p (T3)**	**p-trend (continuous)**
NT-pro-BNP	Fully adjusted	4.4 (-13.4, 26.0)	0.649	-7.2 (-23.3, 12.2)	0.438	0.331
NT-pro-BNP	W/o BMI	4.7 (-13.1, 26.3)	0.627	-6.8 (-22.9, 12.7)	0.467	0.354
NT-pro-BNP	W/o erMedDiet	4.5 (-13.3, 25.9)	0.645	-7.3 (-23.2, 12.0)	0.433	0.317
NT-pro-BNP	W/o BMI and erMedDiet	4.7 (-13.1, 26.2)	0.626	-6.8 (-22.8, 12.5)	0.464	0.343
hs-TnT	Fully adjusted	0.9 (-7.5, 10.0)	0.842	-1.7 (-10.0, 7.3)	0.696	0.674
hs-TnT	W/o BMI	0.6 (-7.8, 9.7)	0.900	-2.3 (-10.5, 6.8)	0.613	0.597
hs-TnT	W/o erMedDiet	0.4 (-7.9, 9.5)	0.921	-1.2 (-9.5, 7.8)	0.782	0.763
hs-TnT	W/o BMI and erMedDiet	0.2 (-8.2, 9.2)	0.970	-1.8 (-10.0, 7.2)	0.687	0.669
CRP	Fully adjusted	-6.0 (-23.8, 16.1)	0.566	-6.1 (-24.2, 16.3)	0.563	0.639
CRP	W/o BMI	-7.3 (-25.2, 14.9)	0.488	-8.3 (-26.2, 14.1)	0.437	0.509
CRP	W/o erMedDiet	-5.1 (-23.0, 17.1)	0.628	-7.2 (-24.9, 14.8)	0.493	0.538
CRP	W/o BMI and erMedDiet	-6.2 (-24.3, 16.1)	0.556	-9.6 (-27.2, 12.3)	0.363	0.401
PICP	Fully adjusted	-0.6 (-9.9, 9.7)	0.904	-4.0 (-13.1, 6.1)	0.421	0.412
PICP	W/o BMI	-0.7 (-10.0, 9.5)	0.884	-4.2 (-13.3, 5.8)	0.397	0.389
PICP	W/o erMedDiet	-0.9 (-10.1, 9.3)	0.854	-3.7 (-12.7, 6.4)	0.462	0.452
PICP	W/o BMI and erMedDiet	-1.0 (-10.2, 9.1)	0.837	-3.9 (-12.9, 6.1)	0.434	0.425
3-NT	Fully adjusted	-4.9 (-21.5, 15.1)	0.604	5.4 (-13.1, 28.0)	0.593	0.473
3-NT	W/o BMI	-4.4 (-21.1, 15.7)	0.644	6.3 (-12.4, 29.1)	0.535	0.424
3-NT	W/o erMedDiet	-3.3 (-20.1, 17.0)	0.728	3.4 (-14.8, 25.4)	0.734	0.633
3-NT	W/o BMI and erMedDiet	-2.9 (-19.8, 17.5)	0.762	4.4 (-14.0, 26.6)	0.666	0.570

Percent difference (95% CI) in ln(biomarker) across tertiles of non-marine omega-3 fatty acid intake. T1 = reference.

Fully adjusted model adjusted for total energy intake (kcal/day), sex, age (years), intervention group, prevalent type 2 diabetes, prevalent cancer, smoking status (never / former / current), marital status, hypercholesterolemia, hypertension, sleep apnea, leisure-time physical activity (METs·min/week), body-mass index (kg/m²), 17-item erMedDiet adherence score, estimated glomerular filtration rate (mL/min/1.73 m²) and educational level.

Sensitivity specifications were identical to the fully adjusted model except as follows:

- **No-BMI model**: body-mass index removed.
- **No-erMedDiet model**: 17-item erMedDiet adherence score removed.
- **No-both model**: both body-mass index and the 17-item erMedDiet adherence score removed.

3-NT, 3-nitrotyrosine; BMI, body-mass index; CI, confidence interval; CRP, C-reactive protein (high-sensitivity); eGFR, estimated glomerular filtration rate; erMedDiet, adherence to the energy-reduced Mediterranean diet; hs-TnT, high-sensitivity cardiac troponin T; METs, metabolic equivalents of task; NT-pro-BNP, N-terminal pro–B-type natriuretic peptide; PICP, procollagen type I C-terminal propeptide; T1/T2/T3, tertile 1 / tertile 2 / tertile 3 of exposure (T1 = reference); W/o, without.

Supplemental Table S2f. Sensitivity analyses to assess potential overadjustment for basal non-marine omega-3 fatty acid intake (longitudinal mixed models)

Biomarker	Specification	T1: Y3-Y0 %diff (95% CI)	T1: Y5-Y0 %diff (95% CI)	T2: Y3-Y0 %diff (95% CI)	T2: Y5-Y0 %diff (95% CI)	T3: Y3-Y0 %diff (95% CI)	T3: Y5-Y0 %diff (95% CI)	p-interaction (tertile#visit, Wald)
NT-pro-BNP	Fully adjusted	8.1 (-5.6, 23.8)	25.3 (9.2, 43.8)	9.6 (-4.3, 25.6)	20.9 (5.1, 39.2)	21.5 (7.1, 37.8)	28.7 (13.0, 46.7)	0.619
NT-pro-BNP	W/o BMI	8.1 (-5.6, 23.8)	25.3 (9.2, 43.8)	9.7 (-4.3, 25.6)	20.9 (5.1, 39.2)	21.5 (7.1, 37.8)	28.8 (13.0, 46.7)	0.620
NT-pro-BNP	W/o erMedDiet	13.7 (1.0, 28.0)	31.7 (16.7, 48.6)	15.7 (2.9, 30.0)	27.6 (13.1, 44.1)	25.4 (11.5, 40.9)	33.9 (18.7, 51.0)	0.754
NT-pro-BNP	W/o BMI and erMedDiet	13.7 (1.0, 28.0)	31.7 (16.7, 48.6)	15.7 (2.9, 30.0)	27.6 (13.1, 44.1)	25.4 (11.5, 40.9)	33.9 (18.7, 51.0)	0.754
hs-TnT	Fully adjusted	8.3 (3.1, 13.7)	16.8 (11.1, 22.8)	10.1 (4.8, 15.7)	18.1 (12.2, 24.3)	10.5 (5.5, 15.6)	19.5 (13.9, 25.3)	0.938
hs-TnT	W/o BMI	8.4 (3.2, 13.9)	16.9 (11.2, 22.9)	10.3 (4.9, 15.8)	18.2 (12.3, 24.4)	10.5 (5.6, 15.7)	19.6 (14.0, 25.4)	0.941
hs-TnT	W/o erMedDiet	8.9 (4.3, 13.6)	17.4 (12.4, 22.6)	10.7 (6.1, 15.4)	18.8 (13.7, 24.1)	11.0 (6.4, 15.8)	20.0 (14.9, 25.3)	0.948
hs-TnT	W/o BMI and erMedDiet	8.9 (4.4, 13.7)	17.5 (12.5, 22.7)	10.7 (6.2, 15.5)	18.8 (13.7, 24.1)	11.0 (6.4, 15.8)	20.0 (14.9, 25.4)	0.950
CRP	Fully adjusted	-4.3 (-18.1, 11.7)	-15.9 (-28.1, -1.6)	3.9 (-11.1, 21.3)	-8.7 (-22.2, 7.2)	-15.7 (-27.0, -2.6)	-17.0 (-28.5, -3.6)	0.280
CRP	W/o BMI	-3.4 (-17.3, 12.8)	-15.1 (-27.4, -0.6)	4.8 (-10.3, 22.5)	-8.0 (-21.7, 8.0)	-15.4 (-26.8, -2.2)	-16.5 (-28.1, -3.0)	0.258
CRP	W/o erMedDiet	-5.6 (-17.6, 8.1)	-17.0 (-27.8, -4.7)	2.1 (-10.7, 16.8)	-10.0 (-21.7, 3.4)	-16.2 (-26.8, -4.2)	-17.9 (-28.4, -5.7)	0.320
CRP	W/o BMI and erMedDiet	-5.1 (-17.1, 8.7)	-16.5 (-27.3, -4.1)	2.6 (-10.3, 17.4)	-9.8 (-21.5, 3.7)	-16.1 (-26.7, -4.0)	-17.6 (-28.2, -5.4)	0.302
PICP	Fully adjusted	5.1 (-3.0, 13.8)	8.1 (-0.3, 17.2)	-4.4 (-11.7, 3.5)	-9.2 (-16.4, -1.4)	-0.5 (-7.6, 7.2)	-2.5 (-9.7, 5.3)	0.017
PICP	W/o BMI	5.0 (-3.0, 13.7)	8.1 (-0.3, 17.1)	-4.5 (-11.8, 3.5)	-9.2 (-16.4, -1.4)	-0.5 (-7.6, 7.1)	-2.5 (-9.7, 5.3)	0.017
PICP	W/o erMedDiet	4.4 (-2.7, 11.9)	7.4 (0.0, 15.3)	-5.0 (-11.4, 1.8)	-9.8 (-16.0, -3.1)	-1.0 (-7.7, 6.0)	-3.0 (-9.6, 4.1)	0.016
PICP	W/o BMI and erMedDiet	4.3 (-2.7, 11.9)	7.4 (-0.0, 15.3)	-5.1 (-11.4, 1.7)	-9.8 (-16.0, -3.1)	-1.0 (-7.7, 6.0)	-3.0 (-9.6, 4.1)	0.017
3-NT	Fully adjusted	6.1 (-5.9, 19.6)	9.8 (-2.7, 24.0)	14.7 (1.7, 29.3)	15.6 (2.1, 30.9)	14.2 (2.2, 27.6)	22.1 (8.8, 37.0)	0.601
3-NT	W/o BMI	6.0 (-6.0, 19.5)	9.7 (-2.8, 23.9)	14.6 (1.6, 29.2)	15.5 (2.0, 30.8)	14.2 (2.2, 27.6)	22.0 (8.7, 36.9)	0.599
3-NT	W/o erMedDiet	1.7 (-8.3, 12.8)	5.4 (-5.2, 17.2)	9.8 (-0.9, 21.7)	10.6 (-0.6, 23.0)	10.9 (0.1, 22.9)	18.2 (6.4, 31.4)	0.528
3-NT	W/o BMI and erMedDiet	1.7 (-8.3, 12.8)	5.3 (-5.3, 17.1)	9.7 (-0.9, 21.6)	10.5 (-0.6, 22.9)	10.9 (0.1, 22.9)	18.2 (6.3, 31.3)	0.526

Percent change (95% CI) in ln(biomarker) at year 3 and year 5 within each tertile of non-marine omega-3 fatty acid intake (vs baseline).

Linear mixed models with random intercepts were used.

Fully adjusted model adjusted for total energy intake (kcal/day), sex, age (years), intervention group, prevalent type 2 diabetes, prevalent cancer, smoking status (never / former / current), marital status, hypercholesterolemia, hypertension, sleep apnea, leisure-time physical activity (METs·min/week), body-mass index (kg/m²), 17-item erMedDiet adherence score, estimated glomerular filtration rate (mL/min/1.73 m²) and educational level.

Sensitivity specifications were identical to the fully adjusted model except as follows:

- **No-BMI model**: body-mass index removed.
- **No-erMedDiet model**: 17-item erMedDiet adherence score removed.
- **No-both model**: both body-mass index and the 17-item erMedDiet adherence score removed.

3-NT, 3-nitrotyrosine; BMI, body-mass index; CI, confidence interval; CRP, C-reactive protein (high-sensitivity); eGFR, estimated glomerular filtration rate; erMedDiet, adherence to the energy-reduced Mediterranean diet; hs-TnT, high-sensitivity cardiac troponin T; METs, metabolic equivalents of task; NT-pro-BNP, N-terminal pro–B-type natriuretic peptide; PICP, procollagen type I C-terminal propeptide; T1/T2/T3, tertile 1 / tertile 2 / tertile 3 of exposure (T1 = reference); W/o, without.

Supplemental Table S3. Comparison of baseline characteristics of retained participants vs participants lost to follow-up

Variable	Total (n=510)	Retained Y3	Lost Y3	p(Y3)	SMD (Y3)	Retained Y5	Lost Y5	p(Y5)	SMD (Y5)
Age, years	65.18 (4.88)	65.19 (4.80)	65.00 (6.25)	0.843	0.034	65.14 (4.82)	65.62 (5.52)	0.524	-0.094
BMI, kg/m^2	32.13 (3.28)	32.16 (3.29)	31.66 (3.06)	0.430	0.159	32.11 (3.27)	32.35 (3.39)	0.642	-0.072
Total energy intake, kcal/d	2370.08 (578.24)	2369.67 (576.57)	2377.10 (617.22)	0.947	-0.012	2394.71 (579.38)	2115.58 (505.28)	0.002	0.513
Physical activity, METs-min/d	2535.92 (2271.01)	2538.58 (2263.20)	2490.03 (2444.84)	0.913	0.021	2562.72 (2319.43)	2258.97 (1686.63)	0.392	0.150
erMedDiet 17-item score	7.72 (2.93)	7.68 (2.93)	8.46 (2.95)	0.167	-0.268	7.72 (2.91)	7.71 (3.17)	0.984	0.003
eGFR, mL/min/1.73 m^2	90.53 (12.04)	90.58 (12.09)	89.59 (11.25)	0.672	0.085	90.49 (12.21)	90.88 (10.18)	0.838	-0.034
Total omega-3 intake, g/d (energy-adjusted)	2.18 (0.75)	2.16 (0.74)	2.44 (0.82)	0.060	-0.351	2.19 (0.76)	2.09 (0.63)	0.381	0.147
Marine omega-3 intake, g/d (energy-adjusted)	0.59 (0.32)	0.58 (0.32)	0.72 (0.34)	0.028	-0.417	0.59 (0.32)	0.61 (0.31)	0.671	-0.068
Non-marine omega-3 intake, g/d (energy-adjusted)	1.59 (0.65)	1.58 (0.65)	1.72 (0.72)	0.284	-0.199	1.60 (0.67)	1.48 (0.51)	0.226	0.209
NT-pro-BNP, pg/mL	77.43 (129.21)	74.43 (106.48)	128.92 (331.44)	0.030	-0.221	72.25 (99.46)	130.86 (292.61)	0.004	-0.268
hs-TnT, ng/L	9.33 (4.77)	9.39 (4.82)	8.24 (3.73)	0.216	0.266	9.39 (4.87)	8.71 (3.59)	0.363	0.159
hs-CRP, mg/dL	0.40 (0.70)	0.41 (0.72)	0.32 (0.34)	0.500	0.164	0.39 (0.69)	0.49 (0.78)	0.400	-0.125
PICP, ng/mL	97.04 (42.18)	96.96 (41.59)	98.47 (52.22)	0.853	-0.032	97.90 (41.54)	88.09 (47.94)	0.136	0.219
3-nitrotyrosine (3-NT), nmol/L	752.27 (683.23)	748.25 (675.80)	821.38 (811.68)	0.582	-0.098	756.41 (683.31)	709.48 (688.59)	0.660	0.068
Women, %	40.6%	40.5% (n=482)	42.9% (n=28)	0.801	-0.049	39.8% (n=465)	48.9% (n=45)	0.235	-0.184
Type 2 diabetes, %	28.8%	28.6% (n=482)	32.1% (n=28)	0.690	-0.076	28.8% (n=465)	28.9% (n=45)	0.992	-0.002
Hypertension, %	87.6%	87.8% (n=482)	85.7% (n=28)	0.749	0.060	87.3% (n=465)	91.1% (n=45)	0.460	-0.123
Dyslipidemia, %	73.9%	74.7% (n=482)	60.7% (n=28)	0.102	0.302	74.4% (n=465)	68.9% (n=45)	0.421	0.123
Cancer at baseline, %	7.5%	7.3% (n=482)	10.7% (n=28)	0.499	-0.121	6.7% (n=465)	15.6% (n=45)	0.030	-0.286
Sleep apnea, %	16.1%	16.4% (n=482)	10.7% (n=28)	0.427	0.166	16.1% (n=465)	15.6% (n=45)	0.920	0.016
Intervention group (intensive), %	51.0%	50.6% (n=482)	57.1% (n=28)	0.502	-0.131	50.1% (n=465)	60.0% (n=45)	0.205	-0.200
≥Secondary education, %	80.0%	80.1% (n=482)	78.6% (n=28)	0.846	0.037	80.2% (n=465)	77.8% (n=45)	0.696	0.060
Smoking status (chi^2 global)				0.221				0.403	
- Never smoker	39.6%	38.8%	53.6%		−0.3%	39.1%	44.4%		−0.11%
- Current smoker	10.0%	10.4%	3.6%		0.27%	10.5%	4.4%		0.23%
- Former smoker	50.4%	50.8%	42.9%		0.16%	50.3%	51.1%		−0.02%
Marital status (chi^2 global)				0.950				0.201	
- Married	78.4%	78.4%	78.6%		0 %	79.6%	66.7%		0.30%
- Single	5.7%	5.6%	7.1%		−0.06%	5.4%	8.9%		-0.13%
- Widowed	10.2%	10.2%	10.7%		−0.02%	9.5%	17.8%		-0.24%
- Separated / divorced	5.7%	5.8%	3.6%		0.1%	5.6%	6.7%		-0.04%

Mean (SD) for continuous variables; n (%) for categorical variables. SMD = standardized mean difference; |SMD|<0.10 balanced, 0.10–0.20 mild, ≥0.20 relevant. Smoking and marital status reported as global χ^2 p-value plus per-category breakdown. N/A: SMD does not apply to qualitative variables.

Supplemental Table S4a. IPAW-weighted vs unweighted longitudinal analysis for total omega-3 fatty acid intake

Biomarker	Tertile	Unweighted: Y5-Y0 %diff (95% CI)	p (unw)	IPAW: Y5-Y0 %diff (95% CI)	p (IPAW)	p-interaction (unw)	p-interaction (IPAW)
NT-pro-BNP	T1	24.1 (8.2, 42.3)	0.002	19.4 (0.3, 42.0)	0.046	0.812	0.928
NT-pro-BNP	T2	21.0 (4.8, 39.7)	0.009	15.3 (-5.5, 40.6)	0.161		
NT-pro-BNP	T3	30.0 (14.2, 47.9)	<0.001	20.8 (4.1, 40.0)	0.013		
hs-TnT	T1	16.0 (10.4, 21.9)	<0.001	12.4 (5.5, 19.9)	<0.001	0.786	0.728
hs-TnT	T2	19.2 (13.2, 25.7)	<0.001	16.1 (8.2, 24.7)	<0.001		
hs-TnT	T3	19.0 (13.6, 24.7)	<0.001	13.3 (5.9, 21.3)	<0.001		
CRP	T1	-17.5 (-29.4, -3.5)	0.016	-24.4 (-36.0, -10.8)	<0.001	0.284	0.144
CRP	T2	-6.7 (-20.8, 9.9)	0.408	-10.9 (-26.2, 7.6)	0.230		
CRP	T3	-17.6 (-28.9, -4.6)	0.010	-22.4 (-34.2, -8.5)	0.003		
PICP	T1	1.3 (-6.5, 9.9)	0.745	1.4 (-11.7, 16.4)	0.840	0.705	0.771
PICP	T2	-4.4 (-12.2, 4.0)	0.292	-2.9 (-12.5, 7.7)	0.575		
PICP	T3	-0.5 (-7.8, 7.4)	0.904	14.5 (-10.8, 47.0)	0.286		
3-NT	T1	8.6 (-3.8, 22.5)	0.182	11.5 (-4.0, 29.4)	0.153	0.423	0.487
3-NT	T2	24.1 (9.3, 40.9)	<0.001	26.8 (7.2, 50.1)	0.006		
3-NT	T3	16.7 (4.1, 30.7)	0.008	18.9 (2.1, 38.4)	0.026		

Percent change in biomarkers at year 5 vs baseline within each tertile.

Primary = unweighted; IPAW = inverse probability of attrition weighting.

Supplemental Table S4b. IPAW-weighted vs unweighted longitudinal analysis for marine omega-3 fatty acid intake

Biomarker	Tertile	Unweighted: Y5-Y0 %diff (95% CI)	p (unw)	IPAW: Y5-Y0 %diff (95% CI)	p (IPAW)	p-interaction (unw)	p-interaction (IPAW)
NT-pro-BNP	T1 (Y5-Y0)	25.1 (8.8, 43.9)	0.002	20.5 (1.0, 43.8)	0.038	0.487	0.549
NT-pro-BNP	T2 (Y5-Y0)	27.6 (11.6, 45.9)	<0.001	17.0 (-3.0, 41.2)	0.101		
NT-pro-BNP	T3 (Y5-Y0)	25.4 (9.8, 43.3)	<0.001	19.5 (2.8, 38.9)	0.020		
hs-TnT	T1 (Y5-Y0)	17.9 (12.1, 24.0)	<0.001	14.9 (6.7, 23.7)	<0.001	0.134	0.460
hs-TnT	T2 (Y5-Y0)	22.4 (16.6, 28.5)	<0.001	16.3 (8.7, 24.3)	<0.001		
hs-TnT	T3 (Y5-Y0)	14.6 (9.2, 20.2)	<0.001	10.9 (4.4, 17.7)	<0.001		
CRP	T1 (Y5-Y0)	-20.3 (-32.0, -6.4)	0.005	-26.7 (-38.6, -12.4)	<0.001	0.827	0.579
CRP	T2 (Y5-Y0)	-12.4 (-24.9, 2.1)	0.090	-17.1 (-29.5, -2.4)	0.024		
CRP	T3 (Y5-Y0)	-12.5 (-24.8, 1.9)	0.086	-14.9 (-28.9, 1.9)	0.080		
PICP	T1 (Y5-Y0)	-3.2 (-10.8, 5.1)	0.440	-1.8 (-15.1, 13.5)	0.802	0.500	0.148
PICP	T2 (Y5-Y0)	-1.6 (-9.1, 6.5)	0.685	14.0 (-10.4, 45.0)	0.285		
PICP	T3 (Y5-Y0)	0.6 (-7.0, 8.8)	0.889	-0.1 (-7.2, 7.5)	0.981		
3-NT	T1 (Y5-Y0)	17.8 (4.1, 33.3)	0.009	24.4 (5.2, 47.1)	0.011	0.997	0.981
3-NT	T2 (Y5-Y0)	14.9 (2.1, 29.3)	0.022	19.2 (2.8, 38.2)	0.020		
3-NT	T3 (Y5-Y0)	16.4 (3.5, 30.9)	0.012	17.7 (1.0, 37.1)	0.037		

Percent change in biomarkers at year 5 vs baseline within each tertile.

Primary = unweighted; IPAW = inverse probability of attrition weighting.

Supplemental Table S4c. IPAW-weighted vs unweighted longitudinal analysis for non-marine omega-3 fatty acid intake

Biomarker	Tertile	Unweighted: Y5-Y0 %diff (95% CI)	p (unw)	Cumul IPAW: Y5-Y0 %diff (95% CI)	p (IPAW)	p-interaction (unw)	p-interaction (IPAW)
NT-pro-BNP	T1 (Y5-Y0)	25.3 (9.2, 43.8)	0.001	20.7 (-0.4, 46.3)	0.055	0.619	0.605
NT-pro-BNP	T2 (Y5-Y0)	20.9 (5.1, 39.2)	0.008	15.2 (-3.1, 36.9)	0.109		
NT-pro-BNP	T3 (Y5-Y0)	28.7 (13.0, 46.7)	<0.001	19.5 (2.6, 39.2)	0.022		
hs-TnT	T1 (Y5-Y0)	16.8 (11.1, 22.8)	<0.001	13.0 (5.9, 20.6)	<0.001	0.938	0.969
hs-TnT	T2 (Y5-Y0)	18.1 (12.2, 24.3)	<0.001	15.3 (7.9, 23.3)	<0.001		
hs-TnT	T3 (Y5-Y0)	19.5 (13.9, 25.3)	<0.001	13.6 (5.9, 21.8)	<0.001		
CRP	T1 (Y5-Y0)	-15.9 (-28.1, -1.6)	0.031	-22.7 (-35.3, -7.8)	0.004	0.280	0.130
CRP	T2 (Y5-Y0)	-8.7 (-22.2, 7.2)	0.268	-10.7 (-25.9, 7.5)	0.231		
CRP	T3 (Y5-Y0)	-17.0 (-28.5, -3.6)	0.015	-22.6 (-34.2, -8.9)	0.002		
PICP	T1 (Y5-Y0)	8.1 (-0.3, 17.2)	0.058	7.9 (-4.2, 21.7)	0.211	0.017	0.086
PICP	T2 (Y5-Y0)	-9.2 (-16.4, -1.4)	0.022	-9.1 (-19.1, 2.1)	0.108		
PICP	T3 (Y5-Y0)	-2.5 (-9.7, 5.3)	0.519	12.0 (-12.4, 43.2)	0.365		
3-NT	T1 (Y5-Y0)	9.8 (-2.7, 24.0)	0.129	13.8 (-2.1, 32.4)	0.092	0.601	0.655
3-NT	T2 (Y5-Y0)	15.6 (2.1, 30.9)	0.022	21.8 (3.1, 43.8)	0.021		
3-NT	T3 (Y5-Y0)	22.1 (8.8, 37.0)	<0.001	24.7 (7.2, 45.1)	0.004		

Percent change in biomarkers at year 5 vs baseline within each tertile.

Primary = unweighted; IPAW = inverse probability of attrition weighting.
